# Quantitative Proteomics Revealed the Pharmacodynamic Network of Bugu Shengsui Decoction Promoting Osteoblast Proliferation

**DOI:** 10.3389/fendo.2021.833474

**Published:** 2022-01-25

**Authors:** Xu Wei, Baoyu Qi, Ruyun Ma, Yili Zhang, Ning Liu, Shengjie Fang, Yanning Zhu, Yanming Xie, Jianye Dai, Liguo Zhu

**Affiliations:** ^1^ Wangjing Hospital, China Academy of Chinese Medical Sciences, Beijing, China; ^2^ School of Pharmacy, Lanzhou University, Lanzhou, China; ^3^ School of Traditional Chinese Medicine & School of Integrated Chinese and Western Medicine, Nanjing University of Chinese Medicine, Nanjing, China; ^4^ Institute of Basic Research in Clinical Medicine, China Academy of Chinese Medical Sciences, Beijing, China; ^5^ Collaborative Innovation Center for Northwestern Chinese Medicine, Lanzhou University, Lanzhou, China

**Keywords:** Bugu Shengsui Decoction, PI3K-AKT pathway, osteoporosis, stable isotope dimethyl-labeled proteomics, bone formation

## Abstract

**Background and Objective:**

With high morbidity and disability, osteoporosis is a worldwide bone metabolism disease, regulated by complex pathological processes. Insufficient osteogenesis is greatly essential to osteoporosis. Traditional Chinese Medicine, a complex natural herbal medicine system, has increasingly attracted attention all over the world. Bugu Shengsui Decoction, a compound formula for osteoporosis, has significant clinical effects in the treatment of osteoporosis. Yet the detailed mechanisms are unclear. Thus, we investigated the effects and mechanism of Bugu Shengsui Decoction on osteoporotic rats and osteoblasts *in vitro*.

**Methods:**

In this study, we evaluated the effect of Bugu Shengsui Decoction in an animal model of orchiectomy. Multi-pharmacology indexes revealed that Bugu Shengsui Decoction obviously improved bone metabolism, bone mineral density, bone morphology, and biomechanics in the castrated rats. Then, serum pharmacology was employed to unveil that Bugu Shengsui Decoction promoted the proliferation and differentiation of osteoblasts. Moreover, quantitative proteomics combined with RNA interference assay was used to analyze and verify the pathway and key targets in pro-proliferation of MC3T3-E1 cells.

**Results:**

Bugu Shengsui Decoction obviously improved the worse parameters of bone metabolism, bone mineral density, bone morphology, and biomechanics in a castrated rat model. *In vitro*, Bugu Shengsui Decoction exerted proliferation- and differentiation-promoting effects of osteoblasts induced by serum starvation. Moreover, quantitative proteomics analysis combined with RNA interfere assay illustrated that Bugu Shengsui Decoction promoted osteogenesis *via* the PI3K-AKT pathway.

**Conclusion:**

Summarily, our discoveries certify that Bugu Shengsui Decoction is an effective treatment for osteoporosis *via* PI3K-AKT. This study is not only a beneficial attempt to explore the detailed mechanism of Traditional Chinese formula but also will provide inspiration for the treatment strategy of osteoporosis.

## Introduction

Osteoporosis (OP), a common bone metabolic disease with high incidence, has become one of the urgent global major health problems (1, 2). It is caused by the imbalance of bone metabolism, including bone loss, destruction of bone tissue structure, and increased risk of bone fragility and fractures. It easily leads to disability and mortality, bringing heavy economic and medical burden to society (2). The pathogenesis of OP is complicated, yet more bone resorption than formation is the main pathological manifestation (3). Currently, the anti-osteoporosis strategies include using bone resorption inhibitors, calcium and vitamin supplements, diet adjustments, and increasing physical activities (2–4). Some researchers have demonstrated that the multilevel processes of bone formation are essential for maintaining bone mass (5). Under the regulation of cytokines, ossification centers are formed *via* osteoblasts’ proliferation, differentiation, and migration. It is essential to the formation of bones (6). Therefore, it is beneficial to promote the proliferation, differentiation, and migration of bone cells for the prevention and treatment of OP.

Recently, Traditional Chinese medicine shows obvious curative effects on OP, which significantly increases bone density, reduces the incidence of fragility fractures, and plays anti-inflammatory and analgesic roles in OP patients (7–9). Especially, Bugu Shengsui Decoction (BGSSD), a traditional Chinese medicine compound formula, is applied to treat OP in clinical practice. It can effectively increase bone mineral density (BMD), serum calcitonin, luteinizing hormone, and calcium in OP patients, with total 82% efficiency (10). BGSSD can also improve blood circulation disorders in osteoporotic model rats (11) regulating the expression of related proteins in the Smad/ERK signaling pathway (12). Further, the active ingredients in BGSSD are capable of improving blood circulation in osteoporotic rats, performing anti-inflammatory and analgesic effects, increasing BMD, and regulating gene expressions (13–16). However, the detailed anti-osteoporotic mechanism of BGSSD is still unclear.

**Figure 1:**
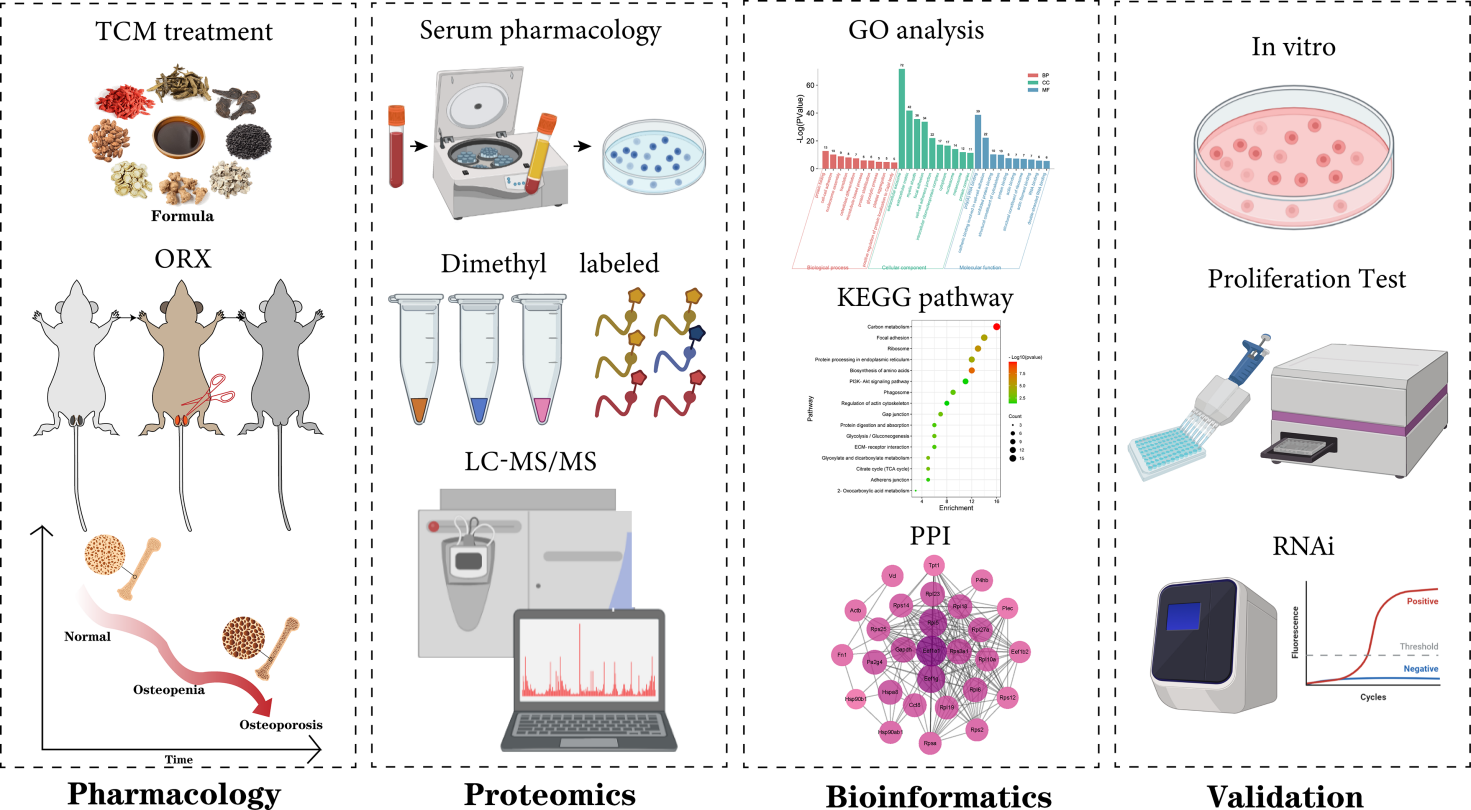
The strategies of revealing the anti-osteoporosis mechanism of BGSSD.

Therefore, we evaluated the anti-osteoporotic effect of BGSSD and explored the specific cellular phenotype (17). Then, a pharmacodynamic network was conducted to analyze the result of quantitative proteomics based on stable isotope dimethyl labeling (18–20). Finally, molecular biology was employed to verify the proposed mechanism of alleviating osteoporosis, relying on the PI3K-AKT pathway. The scheme of this work is as follows (**Figure 1**).

## Materials and Methods

### Animal Experiment

Male Wistar rats (8 weeks old, average weight 244.5 g) were purchased from the Laboratory Animal Center of Lanzhou Veterinary Research Institute, Chinese Academy of Agricultural Sciences, with the license number of SCXK(Gan)2020-0002, raised in the Animal Experiment Center of Lanzhou University, replenished with free food and water. Before modeling, they were fed to adapt to the environment for 7 days. The rats were randomly divided into 6 groups (8 rats in each group), which respectively were groups of control (Ctrl), model (Mod), positive control (P-Ctrl, alendronate sodium), low dose of BGSSD (BGSSD-L), medium dose of BGSSD (BGSSD-M), and high dose of BGSSD (BGSSD-H). A castrated rat model was established to evaluate the anti-osteoporotic effects. Intraperitoneal anesthesia was performed with ethyl ether, and an aseptic modeling operation was performed according to the method of removing testicles (21). Briefly, the midline skin and subcutaneous tissue of the rat scrotum were cut, the testis, epididymis, and surrounding tissues were separated, the testis and epididymis were excised, then the skin incision was sutured. The control group did not do the above operation. In the first 3 days after the operation, rats were injected with penicillin potassium at 50000U per 100g intramuscularly to prevent infection. One week after recovery, saline was given to the control group and model group, alendronate sodium solution (Savio Industrial S.R.L, Italy) was given to the positive control group at 0.88 mg·kg^-1^·d^-1^, and the low dose, medium dose, and high dose of BGSSD groups were respectively given at 2.94, 5.87, and 11.74 g·kg^-1^·day^-1^ intragastrically, once a day for 12 weeks. Chinese herbal medicine was purchased from Wangjing Hospital of China Academy of Chinese Medical Sciences. All administered dosages were converted according to the clinical effective dose. When all animals were euthanized, blood and femurs were collected. All animal experiments were conducted under the procedures and guidelines approved by Lanzhou University Institutional Animal Care and Use Committee and were approved by the Animal Experiments Ethics Committee of Lanzhou University.

### Bone Metabolism Indicator Assay

Serum indicators of bone metabolism such as alkaline phosphatase (Alp), calcium, and phosphorus were assayed according to instructions of the commercial kit (Changchun Huili Biotech Co. Ltd., China). Alp was detected by a three-step reaction. Under alkaline conditions, phenyl disodium phosphate was decomposed into free phenol and phosphoric acid by Alp, which was then combined with aminoantipyrine and oxidized to form a red quinone structure. The quinone conjugate and Alp activity showed a linear relationship at 510 nm. Calcium-GBHA was measured at 520 nm to determine serum calcium concentration. The phosphomolybdic acid was measured at 660 nm to determine the concentration of serum inorganic phosphorus. Serum indicators of bone metabolism were quantitatively measured.

### Bone Mineral Density Test

In order to detect the BMD of rats, the right femur of rats was stored at -80°C, and the whole bone was selected as the target region. A dual-energy X-ray scanner (Osteocore, Medilink, France) was used for scanning. Osteocore can automatically aim at the induction area and control quality and periosteum calibration. Cross-sectional images of the samples were collected, and the range of the femur was framed. Bone mineral mass and bone area were analyzed and calculated by Osteocore 3.7.0.0.5 software; finally, the BMD was calculated.

### Bone Tissue Morphology Test

To analyze the morphological structure, the left femurs were fixed with a 3.7% neutral formalin solution for 24 h and scanned by microcomputed tomography (μ-CT, SkyScan 1174, Bruker, Belgium). Scanning conditions were 50 kV, 800 μA, scanning resolution of 12 μm, and 1,304 × 1,024 pixels of vision. The baseline was 1.0 mm below the growth plate on the knee side of the femur, 250 consecutive sections were taken, and the area with a thickness of 3 mm was selected as the ROI for 3D reconstruction. Three-dimensional (3D) image reconstructions were performed by N-RECON software, and the ratio of bone volume to tissue volume (BV/TV), trabecular thickness (Tb.Th), trabecular number (Tb.N), trabecular separation (Tb.Sp), and structural model index (SMI) were calculated by CT-AN software for 3D analysis.

### Bone Tissue Biomechanical Test

To calculate the biomechanical parameters, the femurs were evaluated on a microcomputer-controlled electronic universal testing machine (RGWT-4005, Shenzhen Ragel, China). Detection conditions were 22°C, span was 20 mm, and running speed was 1 mm per min. The starting point was 2N (displacement clearance at the same time). The fracture sign was the force value less than 20% of the maximum. Bone biomechanical parameters such as maximum load, flexural strength, and elastic modulus of femurs were collected for analysis.

### Preparation of Drug-Containing Serum

After the last intragastric administration, the blood of rats was collected and placed at 4°C for 1 h, then centrifuged for 10 min (4°C, 3,000 rpm). The supernatant was collected, and anhydrous ethanol of 4 times the volume of serum was added to precipitate proteins. The suspension was centrifuged for 10 min (4°C, 5,000 rpm) and the supernatant was dried in a vacuum centrifuge concentrator (45°C, 1,600 rpm, 300 min). The powder was stored separately at -80°C. To eliminate individual differences, the drug-containing serum of each group was premixed and concentrated.

### Cell Culture

Osteoblastic progenitor cells (MC3T3-E1, from China Infrastructure of Cell Line Resource, China) were cultured in alpha-modified minimum essential medium eagle (α-MEM, L570KJ, Shanghai Basalmedia Technologies Co., Ltd., China) containing 10% fetal bovine serum (FBS, 10099-141C, Gibco, USA) and 1% penicillin–streptomycin (15140-122, Gibco, USA) and incubated at 37°C and 5% CO_2_. The medium was changed every 2 days.

### MTS Assay

The cell viability was tested *via* an MTS kit (G3581, Promega, USA). MC3T3-E1 cells were inoculated into 96-well plates at 5*10^3^ per well and incubated overnight to adhere, including a control group and experimental groups. After 8 h, the cells were treated with different drug-containing sera. MTS reagent (10%, v/v) was added after 48 h of administration, and the optical density (OD) was detected at 492 nm to measure the cells’ proliferation.

### Western Blot Assay

The proteins were separated by electrophoresis in 10% sodium dodecyl sulfate-polyacrylamide gel (01413/60341, Cwbio, China), and then the separated proteins were transferred to the polyvinylidene fluoride (PVDF) membrane (A29643309, GE Healthcare, Germany). The PVDF membrane was combined with anti-Col-I antibody (PA5-95137, Sigma, USA), anti-Alp antibody (ET1601-21, HUABIO, China), anti-Runx2 antibody (ET1612-47, HUABIO, China), and anti-β-actin antibody (SA00001-1, Proteintech, USA) incubated overnight at 4°C, then shaken with secondary antibody at room temperature for 1 h. Exposure was taken in a TANON gel imager.

### RNA Interference Assay

MC3T3-E1 cells were plated at 4.0*10^5^ in 6-well plates. The cells with nearly 60% confluency were transfected with siRNA of Fn1, Col1a1, Col1a2, Col6a1, Col6a2, Col6a3, Rac1, Hsp90ab1, Hsp90b1, Ywhaz, PI3K, AKT, BAD, and Bcl-2. Non-specific control (NC) of siRNA was also used. The sequences are shown in **Table 1**.

**Table 1:** Sequences of siRNA used in RNA interference.

siRNA for RNA interference (5′–3′)		
Fn1	Sense	GCCCUAAAGAUUCCAUGAUTT
	Antisense	AUCAUGGAAUCUUUAGGGCTT
Col1a1	Sense	CCCGGAAGAAUACGUAUCATT
	Antisense	UGAUACGUAUUCUUCCGGGTT
Col1a2	Sense	GCCUAGCAACAUGCCAAUATT
	Antisense	UAUUGGCAUGUUGCUAGGCTT
Col6a1	Sense	GUCUGGAAGAUGCAGUAAATT
	Antisense	UUUACUGCAUCUUCCAGACTT
Col6a2	Sense	CCACCACUGAAAGGAACAATT
	Antisense	UUGUUCCUUUCAGUGGUGGTT
Col6a3	Sense	CGGCUGACAUAGUGUUUCUTT
	Antisense	AGAAACACUAUGUCAGCCGTT
Rac1	Sense	GGUGGGAGACGGAGCUGUUTT
	Antisense	AACAGCUCCGUCUCCCACCTT
Hsp90ab1	Sense	CUGGGAACCAUUGCUAAGUTT
	Antisense	ACUUAGCAAUGGUUCCCAGTT
Hsp90b1	Sense	GGUCGUGGAACAACAAUUATT
	Antisense	UAAUUGUUGUUCCACGACCTT
Ywhaz	Sense	CCUUCUCUCUGUUGCUUAUTT
	Antisense	AUAAGCAACAGAGAGAAGGTT
PI3K	Sense	GCAGGAUCAAGUUGUCAAATT
	Antisense	UUUGACAACUUGAUCCUGCTT
AKT	Sense	GGCCCAACACCUUUAUCAUTT
	Antisense	AUGAUAAAGGUGUUGGGCCTT
BAD	Sense	GGAGCAACAUUCAUCAGCATT
	Antisense	UGCUGAUGAAUGUUGCUCCTT
Bcl-2	Sense	GGGAGAUCGUGAUGAAGUATT
	Antisense	UACUUCAUCACGAUCUCCCTT
NC	Sense	UUCUCCGAACGUGUCACGUTT
	Antisense	ACGUGACACGUUCGGAGAATT

All sequences were designed, synthesized, and validated by GenePharma (Shanghai, China). The interference was performed based on the protocol of Lipofectamine^®^ 3000 (2304349, Thermo, USA). The silencing efficiency was examined by qRT-PCR.

### Quantitative Real-Time Polymerase Chain Reaction

Total RNA was extracted and purified according to the commercial kit (LS1040, Promega, USA) and used as a template for cDNA reverse transcription synthesis with the commercial kit (K1622, Thermo, USA). The conditions were as follows: 42°C 30 min and 85°C 10 min. By using UltraSYBR Mixture (Low ROX, CW2601, Cwbio, China), quantitative real-time polymerase chain reaction (qRT-PCR) was performed by Stratagene Mx3000P (Agilent, USA); reaction conditions were 95°C 3 min, 95°C 15 s, 55°C 30 s, 72°C 30 s, operated in 40 cycles. The primer gene sequences are listed in **Table 2**.

**Table 2:** Sequences of primers used in qRT-PCR.

Primers for qRT-PCR (5′–3′)		
Fn1	F	CTTGCACGATGATATGGAGA
	R	AGCTGAACACTGGGTGCTAT
Col1a1	F	TCCGGCTCCTGCTCCTCTTA
	R	GTATGCAGCTGACTTCAGGGATGT
Col1a2	F	TCGTGCCTAGCAACATGCC
	R	TTTGTCAGAATACTGAGCAGCAA
Col6a1	F	GATGAGGGTGAAGTGGGAG
	R	CACTCACAGCAGGAGCACAT
Col6a2	F	GATGACATGGAAGACGTCCTTTG
	R	GCTCTGTTTGGCAGGGAAGTT
Col6a3	F	CCTAACCACATATGTTAGTGGAGGT
	R	GAATGTCTCGCTTGCTCTCTG
Rac1	F	CTGCCAATGTTATGGTAGATGG
	R	TTTCAAATGATGCAGGACTCAC
Hsp90ab1	F	TTGACATCATCCCCAACCCTC
	R	ACCAAACTGCCCAATCATGGA
Hsp90b1	F	TTCTGGAAGGAGTTCGGCAC
	R	TCCATGTTGCCAGACCATCC
Ywhaz	F	TAGGTCATCGTGGAGGGTCG
	R	GAAGCATTGGGGATCAAGAACTT
PI3K	F	GATTTGCCCCACCATGACGAGAAGA
	R	CTCCTTCAGGGAGCTGTACAGGTTGTAG
AKT	F	TGTATGAGAAGAAGCTGAGCCC
	R	TCACTGTCCACACACTCCATG
BAD	F	TGAGCCGAGTGAGCAGGAA
	R	GCCTCCATGATGACTGTTGGT
Bcl-2	F	AGTTCGGTGGGGTCATGTGTG
	R	CCAGGTATGCACCCAGAGTG
β-actin	F	TGGCTCCTAGCACCATGAAG
	R	AACGCAGCTCAGTAACAGTCC

All primers were designed and synthesized by SunBiotech (Tianjin, China). Cycle threshold (Ct) values were processed to evaluate the relative mRNA expression via the 2^-ΔΔCt^ method, and β-actin served as the internal control.

### Stable Isotope Dimethyl Labeling

MC3T3-E1 cells were collected after being washed with PBS for 3 times and centrifuged at 4°C, 3,000 rpm for 3 min. The cells were lysed in 0.1% Triton X-100 (T8787, Sigma, USA)–100 mM TEAB (T7408, Sigma, USA) containing EDTA-free protease inhibitor cocktail (04693132001, Sigma-Aldrich, Gemany) by complete ultrasonication on ice. The supernatant was collected, and protein content was detected by Pierce™ BCA Protein Assay Kit. 10-μl samples (3 mg/ml) were reacted with 30 μl 8 M urea and 2 μl 200 mM DTT at 65°C in darkness for 15 min. Then, 2 μl 400 mM iodoacetamide (IAA) was added for reaction for 30 min in darkness at 35°C. 2 μl 200 mM DTT was added to react with the leftover IAA for 15 min at 65°C. 100 μl 100 mM TEAB, 2 μl 0.2 μg/μl trypsin (V528A, Promega, USA), and 1.5 μl 100 mM calcium chloride were added to perform enzyme cutting at 37°C for no less than 14 h. Dimethyl labeling (18) was as follows: for the control (“light”) group, the peptides were reacted with 6 μl 4% CH_2_O (F1635, Sigma, USA) and 6 μl 0.6 M NaBH_3_CN (42077, Sigma, USA); for the modeling (“middle”) group, the peptides were reacted with 6 μl 4% ^13^CD_2_O (596388, Sigma, USA) and 6 μl 0.6 M NaBH_3_CN; and for the BGSSD (“heavy”) group, the peptides were reacted with 6 μl 4% ^13^CD_2_O and 6 μl 0.6 M NaBD_3_CN (190020, Sigma, USA). The labeling reaction was incubated for 1 h at 22°C. It was quenched by 24 μl 1% ammonia hydroxide and 12 μl formic acid. Finally, the “light,” “middle,” and “heavy” samples were mixed and the desalting was performed with Pierce™ C18 Tips (87782, Thermo, USA).

### LC-MS/MS Test and Data Analysis

All samples were analyzed by LC-MS/MS on Q Exactive Orbitrap mass spectrometers (Thermo, USA), coupled with an UltiMate 3000 LC system (22). In short, full-scan mass spectra were captured over the ratio of m/z from 350 to 1,800, using the analyzer with a mass resolution of 7,000 in positive-ion mode. MS/MS fragmentation was performed in a data-dependent mode, of which the TOP 20 intensity ions were selected for MS/MS analysis with a resolution of 17500, using the collision mode of HCD. Other important parameters were isolation window of 2.0-m/z units, default charge of 2+, normalized collision energy of 28%, maximum IT of 50 ms, and dynamic exclusion of 20.0 s. Then, data analysis was performed by ProLuCID. The different isotopic modifications (28.0313 and 34.0631 Da, respectively, for light and heavy labeling) were set as static modifications on the N-terminal of a peptide and lysine. The ratios of reductive dimethylation were quantified by the CIMAGE software. Proteins with an average ratio (light/heavy) of more than 1.5 were selected for further GO analysis.

### Statistical Analysis

Statistical Product and Service Solutions (IBM SPSS 25.0) was used to check the data normality and homogeneity of variance by the Kolmogorov–Smirnov test and Levene’s test, respectively. When three or more means were compared for statistical significance, one- or two-way ANOVA was conducted. The two-sided Student’s t-test was conducted when two groups of measurements were examined for statistical significance. All data are presented as mean ± s.e.m.; *p*-value <0.05 is considered statistically significant.

## Results

### BGSSD Can Prevent Osteoporosis in Castrated Rats

**Figure 2:**
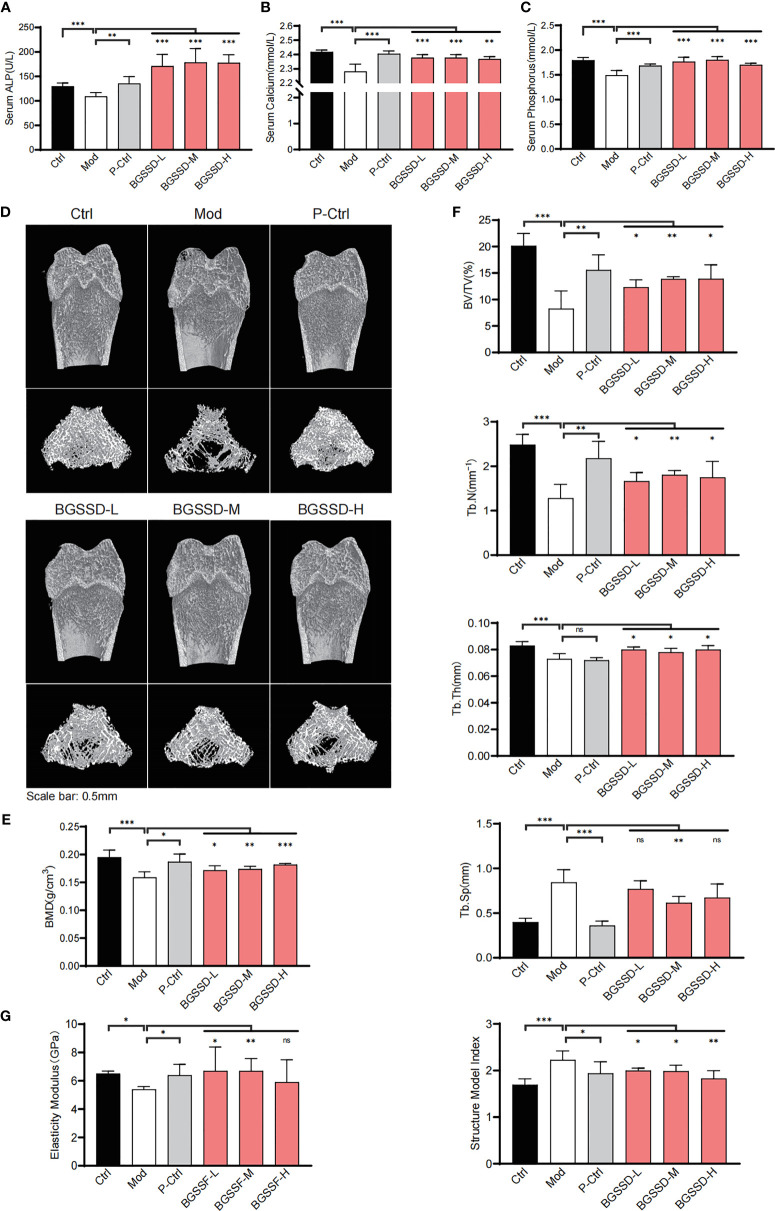
The efficacy results of BGSSD in the treatment of osteoporotic rats. **(A–C)** The effects of different doses of BGSSD on serum Alp, calcium, and phosphorus. **(D)** Representative photomicrographs of distal femur sections by μ-CT. **(E)** Quantitative analysis of BMD (mg·ccm^−1^) of femurs. **(F)** Quantitative analysis of the ratio of bone volume to tissue volume (BV/TV, %), trabecular number (Tb.N, mm^-1^), trabecular thickness (Tb.Th, mm), trabecular separation (Tb.Sp, mm), and the structural model index (SMI) of the μ-CT-scanned distal femurs. **(G)** Quantitative analysis of the elastic modulus of femurs. The results are presented as the mean ± s. e.m., ^*^p < 0.05, ^**^p < 0.01, ^***^ *p* < 0.001, n = 6 per group. NS, not statistically significant.

On the premise of clinical effectiveness, to further determine the therapeutic effects of BGSSD, we established a common orchiectomy-induced (ORX) rat model of OP (21). Littermate male rats received the same bilateral orchiectomy for 12 weeks, and a simultaneous administration was carried out. With the serum markers of bone metabolism detected, we found that the concentrations of Alp, calcium, and phosphorus in serum of ORX rats were significantly decreased. Alendronate as well as three doses of BGSSD could increase the contents of serum Alp, calcium, and phosphorus (**Figures 2A–C**). In order to better quantify and compare the changes of OP, we scanned the femurs *via* μ-CT (**Figure 2D**) and dual-energy X-ray absorptiometry (DXA). Then, we found that the BMD of osteoporotic model rats lessened, and BGSSD can prevent the loss of BMD effectively (**Figure 2E**). Moreover, the microstructure of μ-CT-scanned femurs was further analyzed to demonstrate that ORX rats had a lower percentage of bone volume versus tissue volume (BV/TV), trabecular number (Tb.N), trabecular thickness (Tb.Th), and larger trabecular separation (Tb.Sp) together with the structural model index (SMI) compared with control rats (**Figure 2F**). As expected, BGSSD could ameliorate the microstructural indicators by increasing the ratio of BV/TV, Tb.N, and Tb.Th and decreasing Tb.Sp (medium-dose) and SMI of the ORX rats (**Figure 2F**). In addition, the three-point bending test showed that worse elastic modulus and maximum load are presented in osteoporotic model rats (**Figure 2G** and **Supplementary Material, Figure S1B**), yet BGSSD played a positive role in strengthening elastic modulus (**Figure 2G**). All the above evidence supported that BGSSD is an effective Chinese medical formula for OP.

### BGSSD Can Promote the Proliferation and Differentiation of MC3T3-E1 Cells in Serum-Deprivation-Induced Cell Model

**Figure 3:**
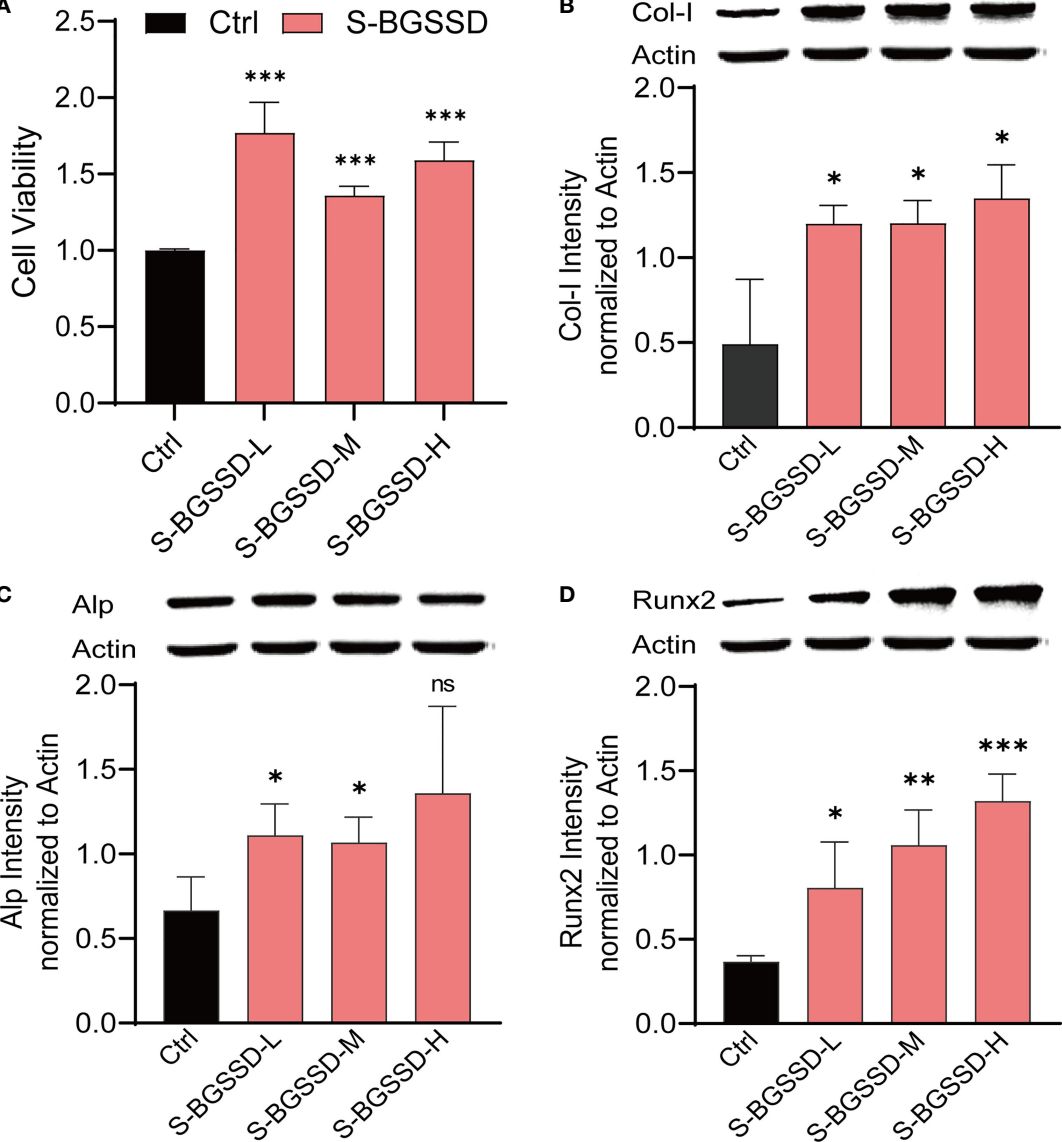
The results of drug-containing serum of BGSSD intervening in the proliferation and differentiation of serum-deprivation-induced MC3T3-E1 cells. **(A)** MTS assay analysis of the viability of MC3T3-E1 cells dealt with the drug-containing serum of low, medium, and high doses of BGSSD (to eliminate the influence of rat serum on cells, all the Ctrl group was treated with the drug-free serum of rats). **(B)** Western blot and quantification (underneath the blots) of Col-I in MC3T3-E1. **(C)** Western blot and quantification (underneath the blots) of Alp in MC3T3-E1. **(D)** Western blot and quantificational analysis (underneath the blots) of Runx2 in MC3T3-E1. Asterisks denote the comparison to serum-starved cells treated with rats’ blank serum (Ctrl). All intervening times of drug-containing serum of BGSSD were 48 h; β-actin served as the internal control in all Western blots. Data are presented as the mean ± s.e.m., ^*^p < 0.05, ^**^p < 0.01, ^***^ *p* < 0.001, n = 3 per group. NS, not statistically significant.

As known, the osteoporotic lesion is in an ischemic and hypoxic environment under the pathological state, which causes the cells to produce a corresponding stress response (23, 24). To further identify the cell phenotype of BGSSD. We screened cell models with the methods of serum deprivation and oxygen deprivation (**Supplementary Material, Figures S3A, B**), and the serum-starved cell model was selected for pharmacodynamic experiments. Finally, we caught that drug-containing serum of BGSSD could obviously increase the viability of serum-deprived MC3T3-E1 cells (**Figure 3A**).

Furthermore, we found that BGGSD could also promote the differentiation of MC3T3-E1 cells. Quantification analysis of Western blots showed that lower expressions of Col-I, Alp, and Runx2 proteins in the Ctrl group and drug-containing serum of all doses of BGSSD could remarkably increase the expressions of Col-I, Alp, and Runx2 (**Figures 3B–D**). The above-listed confirmations indicated that BGSSD could effectively promote both proliferation and differentiation of MC3T3-E1 cells.

### Quantitative Proteomics Revealed the Pharmacodynamic Network and Mechanism of BGSSD

**Figure 4:**
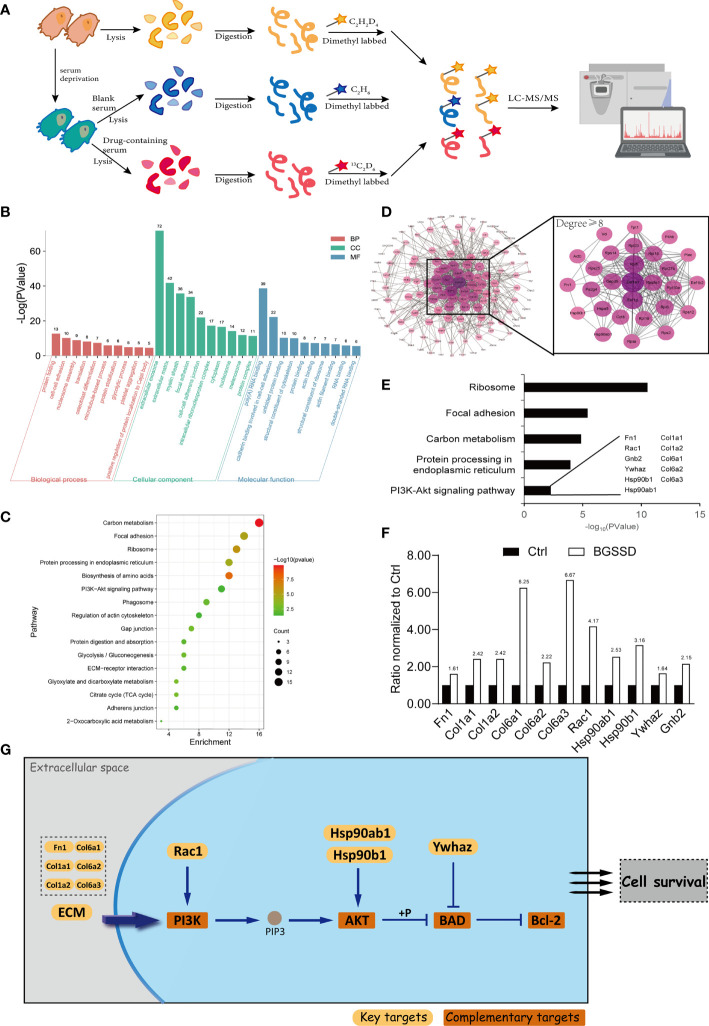
Quantitative proteomics analysis for the pharmacodynamic network and mechanism of BGSSD. **(A)** Quantitative proteomics experimental scheme for detecting the differential expression of proteins. **(B)** Gene Ontology (GO) analysis of the upregulated targets of BGSSD, the top 10 biological processes (BP), cell component (CC), and molecular function (MF) is shown. **(C)** KEGG pathway analysis of the upregulated targets of BGSSD and the top 16 pathways is displayed. **(D)** Protein–protein interaction (PPI) networks of the upregulated targets. The proteins whose degrees are more than 8 were important and enlarged (the right part). **(E)** GO analysis of the important proteins identified by PPI revealed their association with the PI3K-AKT signaling pathway. The key proteins associated with the PI3K-AKT pathway were laid out. **(F)** The expression ratio of key proteins intervened by BGSSD compared to Ctrl. **(G)** The key proteins in the PI3K-AKT pathway.

According to the clinical and experimental effects, quantitative proteomics was conducted to elaborate the pharmacology network and mechanism of BGSSD. The proteomes of the control group, modeling group, and BGSSD-treated group were respectively collected. Then, stable isotope dimethyl labeling was performed to distinguish and simultaneously compare the changes of proteomic networks in three groups (**Figure 4A**).

After bioinformatics analysis (**Figures 4B–D**), GO and KEGG analyses revealed that the top five pathways named ribosome, focal adhesion, carbon metabolism, protein processing in the endoplasmic reticulum, and PI3K-AKT signaling pathway were most relevant to the anti-osteoporosis efficacy of BGSSD (**Figure 4E**). In further literature mining, PI3K-AKT signaling pathway attracted our attention (25), which was highly correlated with the regulation of both proliferation and differentiation of osteoblasts according to published reports (26, 27). Important pharmacodynamic-related proteins in the PI3K-AKT pathway are listed in **Figure 4G**. Fn1, Col1a1, Col1a2, Col6a1, Col6a2, Col6a3, Rac1, Hsp90ab1, Hsp90b1, Ywhaz, and Gnb2 were significantly intervened by BGSSD (**Figure 4F**). Compared with the Ctrl, all the abovementioned proteins were increased in the BGSSD group, which was the content we will focus on especially.

### RNA Interference Verified That the PI3K-AKT Signaling Pathway Are Crucial for the Osteogenetic Effect of BGSSD

**Figure 5:**
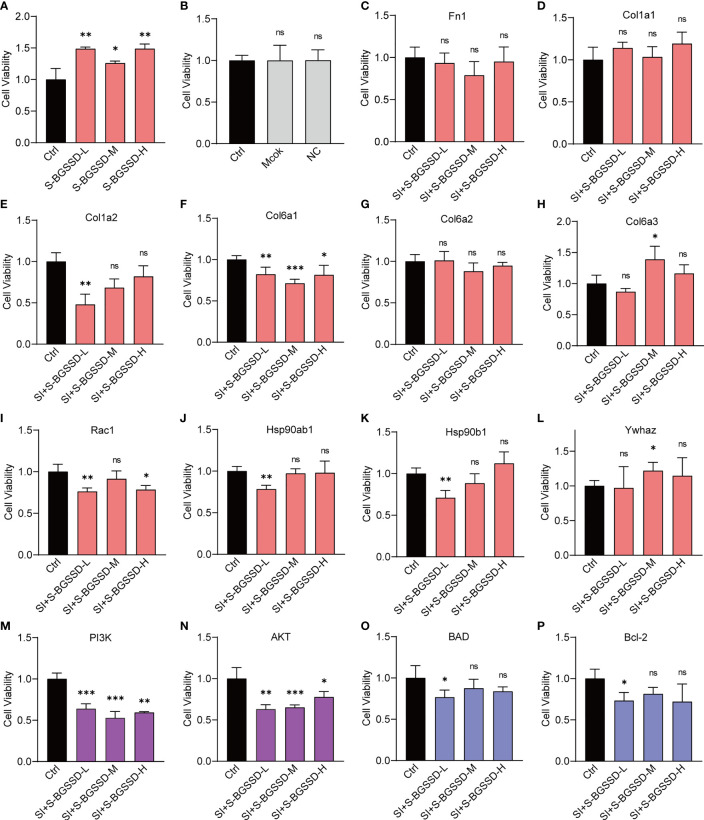
RNA interference to verify the effects of targeting proteins of the PI3K-AKT pathway on the efficacy of BGSSD. **(A)** BGSSD could promote the proliferation of MC3T3-E1 cells. **(B)** The solvent (Mock) and negative control (NC) of siRNA almost had no effect on the viability of MC3T3-E1 cells. **(C–L)** Knockdown of each of the ten targeted proteins (Fn1, Col1a1, Col1a2, Col6a1, Col6a2, Col6a3, Rac1, Hsp90ab1, Hsp90b1, and Ywhaz, except the Col6a3 and Ywhaz in the S-BGSSD-M group) by siRNA abolished the proliferation-promoting effect to various extents of BGSSD in MC3T3-E1 cells. **(M–P)** Knockdown of the proteins of PI3K-AKT and its downstream pathway, named PI3K, AKT, BAD, and Bcl-2, revoked the pro-proliferation effect of BGSSD. The pink columns represent the outcomes of targets obtained by quantitative proteomics. The purple columns refer to the proteins in the PI3K-AKT signaling pathway. The blue are proteins in the downstream pathway. Asterisks denote the comparison to serum-starved cells treated with rats’ blank serum (Ctrl). All intervening times were 48 h. All data are presented as the mean ± s.e.m., ^*^p < 0.05, ^**^p < 0.01, ^***^ *p* < 0.001. NS, not statistically significant.

According to **Figure 5A**, BGSSD could significantly promote the proliferation of serum-deprived MC3T3-E1 cells. With the combination of proteomic analysis and literature mining (28), we found that the PI3K-AKT signaling pathway is closely related to BGSSD’s pharmacological effects, in which the PI3K-AKT pathway played an important role in the process of bone metabolism, which can regulate the proliferation and differentiation of bone formation in the bone formation stage. After comprehensive consideration, Fn1, Col1a1, Col1a2, Col6a1, Col6a2, Col6a3, Rac1, Hsp90ab1, Hsp90b1, Ywhaz, PI3K, AKT, BAD, and Bcl-2 were selected to verify the osteogenetic effect of BGSSD. In this experiment, the solvent (Mock) and negative control (NC) of siRNA almost did not affect the viability of MC3T3-E1 cells (**Figure 5B**), and each of the genes Fn1, Col1a1, Col1a2, Col6a1, Col6a2, Col6a3, Rac1, Hsp90ab1, Hsp90b1, Ywhaz, PI3K, AKT, BAD, and Bcl-2 was effectively knocked down by siRNA (**Supplementary Material, Figures S5A, B**). However, when the above key proteins were knocked down, the pro-proliferation effect of BGSSD was suppressed, even showing an inhibiting effect (**Figures 5C–L**). Among them, the knockdown of Col6a3 and Ywhaz proteins had no significant effect on the proliferation-promoting effect of medium dose (**Figures 5H, L**). Otherwise, we further investigated the functions of other key proteins in PI3K-AKT and its downstream signaling pathway, such as PI3K, AKT, BAD, and Bcl-2, and ascertained that knockdown of the above 4 proteins also eliminated the pro-proliferation effect to a different extent of BGSSD (**Figures 5M–P**).

The outcomes indicate that the PI3K-AKT pathway is indispensable for the pro-proliferation effect of BGSSD.

## Discussion

Osteoporosis is a systemic bone metabolism disease with complex pathogenesis and serious clinical consequences (29). With the aggravation of population aging, the incidence of OP is on the rise. In recent years, extensive studies on the pathogenesis and treatment of OP have revealed that osteoporosis can be prevented by regulating bone metabolism (30–32). It is generally recognized that bone metabolism includes two important processes, which are bone formation mediated by osteoblasts and bone resorption mediated by osteoclasts (32). Previous studies mostly focused on bone resorption, and the clinical drugs approved by the FDA for the treatment of OP are mainly bone resorption inhibitors (2, 33, 34). Currently, only parathyroid hormone-like drugs are approved to promote bone formation, but long-term use of chemical drugs is accompanied by shortcomings of high cost and obvious side effects (2). Therefore, Chinese herbal medicines and compound formulas, used in treating OP for a long time, have received extensive attention and have good research value due to their precise curative effect and few side effects (8, 35).

BGSSD is an effective Chinese medical compound formula for treating OP. Preliminary studies have shown that its clinical efficacy rate is as high as 82%, and it can increase BMD, serum calcitonin, luteinizing hormone, and calcium content (10). Otherwise, Rhizoma Drynariae is the sovereign medicinal in BGSSD, which is a commonly used kidney-tonifying Chinese medicine for the treatment of OP. Among them, flavonoids have a good clinical effect on OP, which can improve lumbar and femoral bone mass density, serum Alp, and other indicators.

Therefore, in order to illustrate the mechanism of BGSSD in treating OP, we designed experiments from multiple perspectives of “formula-herb-molecule.” Firstly, we established a rat model of osteoporosis by removing the testicles and confirmed that BGSSD and Rhizoma Drynariae have similar therapeutic effects on OP, with no significant weight loss (**Supplementary Material, Figures S1A, S2A**). In terms of bone metabolism, Alp is an indicator of bone formation (36); calcium and phosphorus are the “raw materials” for bone formation (37, 38); and BGSSD could effectively increase serum Alp, calcium, and phosphorus concentrations, whereas Rhizoma Drynariae only increased the serum calcium and phosphorus contents distinctly, which indicated that BGSSD and Rhizoma Drynariae may regulate the bone formation process in summary. Furthermore, we found that BGSSD and Rhizoma Drynariae could increase BMD, bone volume to tissue volume (BV/TV), trabecular number (Tb.N), trabecular thickness (Tb.Th), and elastic modulus of femurs and reduce trabecular separation (Tb.Sp) and structural model index (SMI) (**Figures 2A–F**, **Supplementary Material, Figures S2B–G**). However, they have no significant effect on the bending strength and maximum load of the bone (**Supplementary Material, Figures S1B, C, S2H**).

After confirming the efficacy of BGSSD and Rhizoma Drynariae on the model rats, we further studied their effects on MC3T3-E1 cells. To begin, the serum pharmacological method was used to obtain the drug-containing serum with the effective ingredients of BGSSD and Rhizoma Drynariae. The serum-starved cell model was then intervened with drug-containing serum as a therapeutic agent. The results demonstrated that the active ingredients of BGSSD and Rhizoma Drynariae could promote the proliferation and differentiation of MC3T3-E1 cells (**Figure 3A**, **Supplementary Material, Figures S4A, B**).

Relevant studies have also provided some evidence that BGSSD and Rhizoma Drynariae can effectively improve the clinical symptoms related to OP (10, 39, 40). In addition, studies have shown that Rhizoma Drynariae can prevent OP by intervening in oxidative stress response and amino acid metabolism (41). Among them, naringin and naringenin have a two-way regulation of estrogen, which can promote the proliferation of osteoblasts and effectively prevent OP (42). These studies support our experimental results from the side.

Based on previous studies and the abovementioned experimental results, we speculate that BGSSD and the sovereign medicinal Rhizoma Drynariae may play an anti-osteoporotic effect by promoting bone formation. To further prove the speculation, quantitative proteomics strategy and bioinformatics methods were used to analyze the potential drug targets of BGSSD and Rhizoma Drynariae. The results indicated that BGSSD may exert anti-osteoporosis effects through upregulating the pathways of the ribosome, focal adhesion, protein processing in endoplasmic reticulum, carbon metabolism, PI3K-AKT, and so on, and the anti-osteoporosis mechanism of Rhizoma Drynariae may be related to the promotion of functions of ribosome, endoplasmic reticulum, and cell adhesion to a certain extent.

Furthermore, we conducted RNA interference experiments on the important proteins enriched in the PI3K-AKT signaling pathway. Under normal situations, BGSSD can promote the proliferation of MC3T3-E1 cells (**Figure 3A**). When proteins, such as Fn1, Col1a1, Col1a2, Col6a1, Col6a2, Col6a3, Rac1, Hsp90ab1, Hsp90b1, Ywhaz, PI3K, AKT, BAD, and Bcl-2, were knocked down, the effect of BGSSD in promoting the proliferation of MC3T3-E1 cells was suppressed, even showing an opposite effect (**Figures 5C–P**). This evidence indicates that the PI3K-AKT pathway is essential for the anti-osteoporosis of BGSSD, and its key proteins are critical targets for BGSSD to exert anti-osteoporosis effects.

## Conclusion

In this study, we conducted a castrated rat model to evaluate the anti-osteoporotic effects of a traditional Chinese formula, Bugu Shengsui Decoction, which has been proven effective clinically. It is consistent with clinical manifestations that BGSSD presents significant effects in serum markers of bone metabolism, bone mineral density, and tissue morphology. Moreover, these results were derived from that BGSSD can promote the proliferation and differentiation of osteoblastic progenitor cells (MC3T3-E1 cell line). Furthermore, quantitative proteomics with stable isotope dimethyl labeling was employed to unveil the pharmacodynamic network and mechanism of BGSSD. Actually, the PI3K-AKT pathway had been proved to be crucial for the efficacy, of which key proteins’ effects had been verified by RNAi experiments. With the combination of systemic-level, cellular-level, and molecular-level experiments, we could confirm the curative effect, cell phenotype, and molecular mechanism of BGSSD. This work may enlighten a new perspective for exploring the material basis of BGSSD and treatment strategy for osteoporosis *via* the PI3K-AKT pathway.

## Data Availability Statement

The original contributions presented in the study are publicly available. These data can be found here: PRIDE (PRoteomics IDEntifications Database), PXD030275.

## Ethics Statement

The animal study was reviewed and approved by the Animal Ethics Committee, School of Pharmacy, Lanzhou University.

## Author Contributions

XW, YX, JD, and LZ designed and developed the experiments. XW and BQ prepared the draft of the manuscript. BQ, RM, YLZ, NL, SF, and YZ participated in all the experiments. XW, YX, JD, and LZ provided the conditions of experiments. XW, BQ, and RM analyzed the data and drafted the manuscript. YX, JD, and LZ supervised all research and revised the manuscript. All authors contributed to the article and approved the submitted version.

## Funding

This work was supported by the National Natural Science Foundation of China (NSFC) Youth Program (81704102), the Fundamental Research Funds for the Central Public Welfare Research Institutes (ZZ15-XY-CT-06, ZZ13-YQ-039), the Inheritance and Innovation Team Project of National Traditional Chinese Medicine (ZYYCXTD-C-202003), National Key Research and Development Program of China (2018YFC17063005), the Science and Technology Planning Project of Gansu Province (20JR10RA586), the Fundamental Research Funds for the Central Universities (lzujbky-2021-kb40), and the Project for Longyuan Youth Innovation and Entrepreneurship Talent.

## Conflict of Interest

The authors declare that the research was conducted in the absence of any commercial or financial relationships that could be construed as a potential conflict of interest.

## Publisher’s Note

All claims expressed in this article are solely those of the authors and do not necessarily represent those of their affiliated organizations, or those of the publisher, the editors and the reviewers. Any product that may be evaluated in this article, or claim that may be made by its manufacturer, is not guaranteed or endorsed by the publisher.
