# Supplementary material for: Quantitative Proteomics Revealed the Pharmacodynamic Network of Bugu Shengsui Decoction Promoting Osteoblast Proliferation
Source: Front Endocrinol (Lausanne). 2022 Jan 25;12:833474. doi: 10.3389/fendo.2021.833474 (PMC8822948; doi:10.3389/fendo.2021.833474)
Supplement: Supplementary file 1 [file Presentation_1.pdf]

Supplementary Material

This PDF file includes:

Figures S1 to S5 and data availability statement

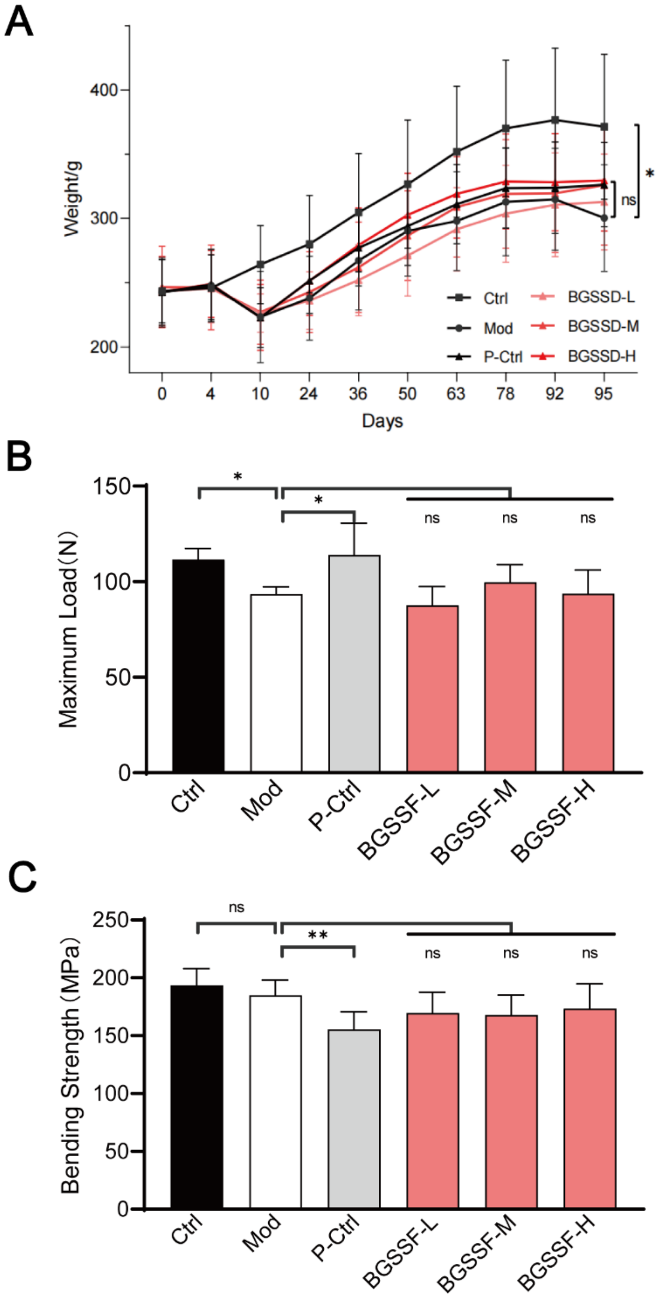

Figure S1. Effects of BGSSD on weight and bone biomechanics of ORX rats. **(A)** The weight of Ctrl was the highest, and there was no significant difference in drug-intervened groups compared with the Mod. **(B)** BGSSD had no significant effect on the maximum load of femurs. **(C)** There was no obvious effect on the bending strength. The results are presented as the mean ±s.e.m., $^{*}P$<0.05, $^{**}P$<0.01, $^{***}P$<0.001, n=6. NS, no significant.

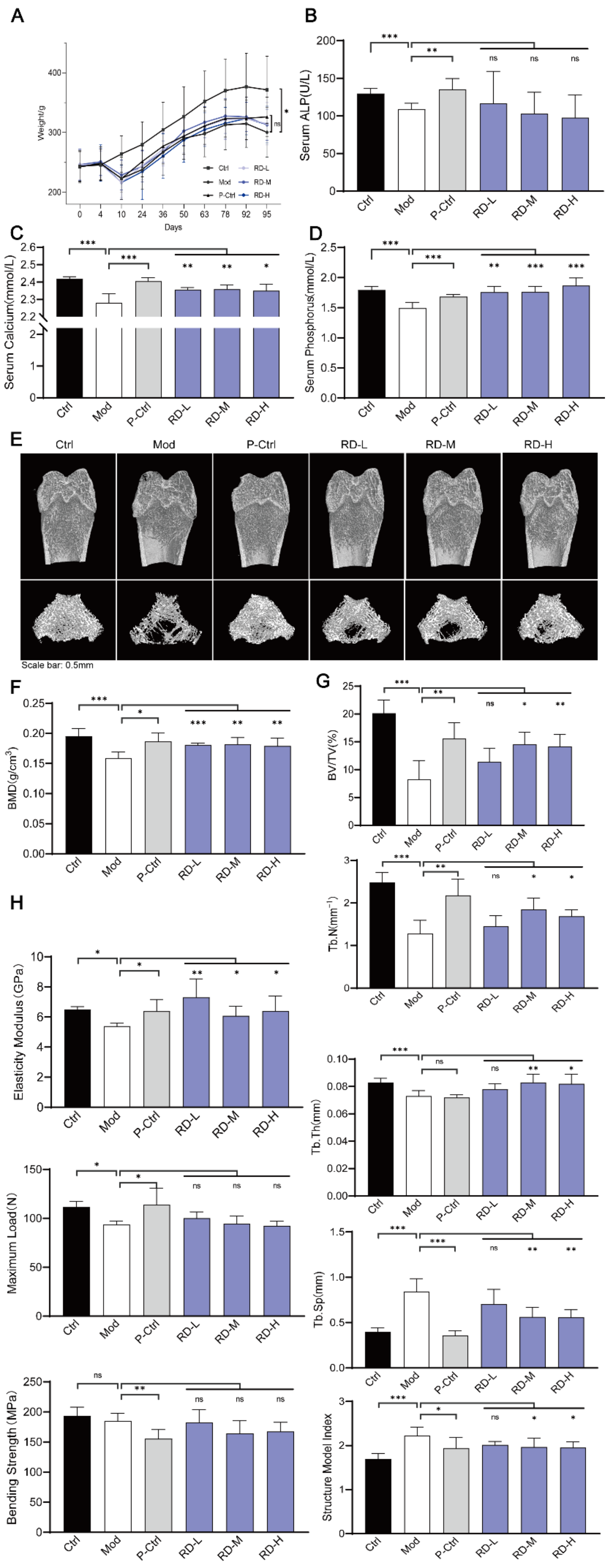
A
B
C
D
E
F
G
H
Weight/g
Days
Ctrl
Mod
P-Ctrl
RD-L
RD-M
RD-H
Serum ALP(U/L)
Serum Calcium(mmol/L)
Serum Phosphorus(mmol/L)
Scale bar: 0.5mm
BMD(g/cm³)
BV/TV(%)
Tb.N(mm⁻¹)
Tb.Th(mm)
Tb.Sp(mm)
Structure Model Index
Elasticity Modulus (GPa)
Maximum Load(N)
Bending Strength (MPa)

Figure S2. The effects of Rhizoma Drynariae on weight, bone metabolism indicators, bone mineral density, tissue morphology and biomechanics of femurs in ORX rats. **(A)** The weight of Ctrl was the highest, and there was no significant difference in drug-intervened groups compared with the Mod. **(B-D)** The functions of Rhizoma Drynariae on serum ALP, calcium and phosphorus. **(E)** Representative photomicrographs of distal femur sections by μ-CT. **(F)** Quantitative analysis of BMD ($mg{\cdot}ccm^{-1}$) of femurs. **(G)** Quantitative analysis of the ratio of bone volume to tissue volume (BV/TV, %), trabecular number (Tb.N, mm-1), trabecular thickness (Tb.Th, mm), trabecular separation (Tb.Sp, mm) and the structural model index (SMI) of the μ-CT-scanned distal femurs. **(H)** Quantitative analysis of the biomechanics of femurs. The results are presented as the mean ±s.e.m., $^{*}P<0.05$, $^{**}P<0.01$, $^{***}P<0.001$, n=6. NS, no significant.

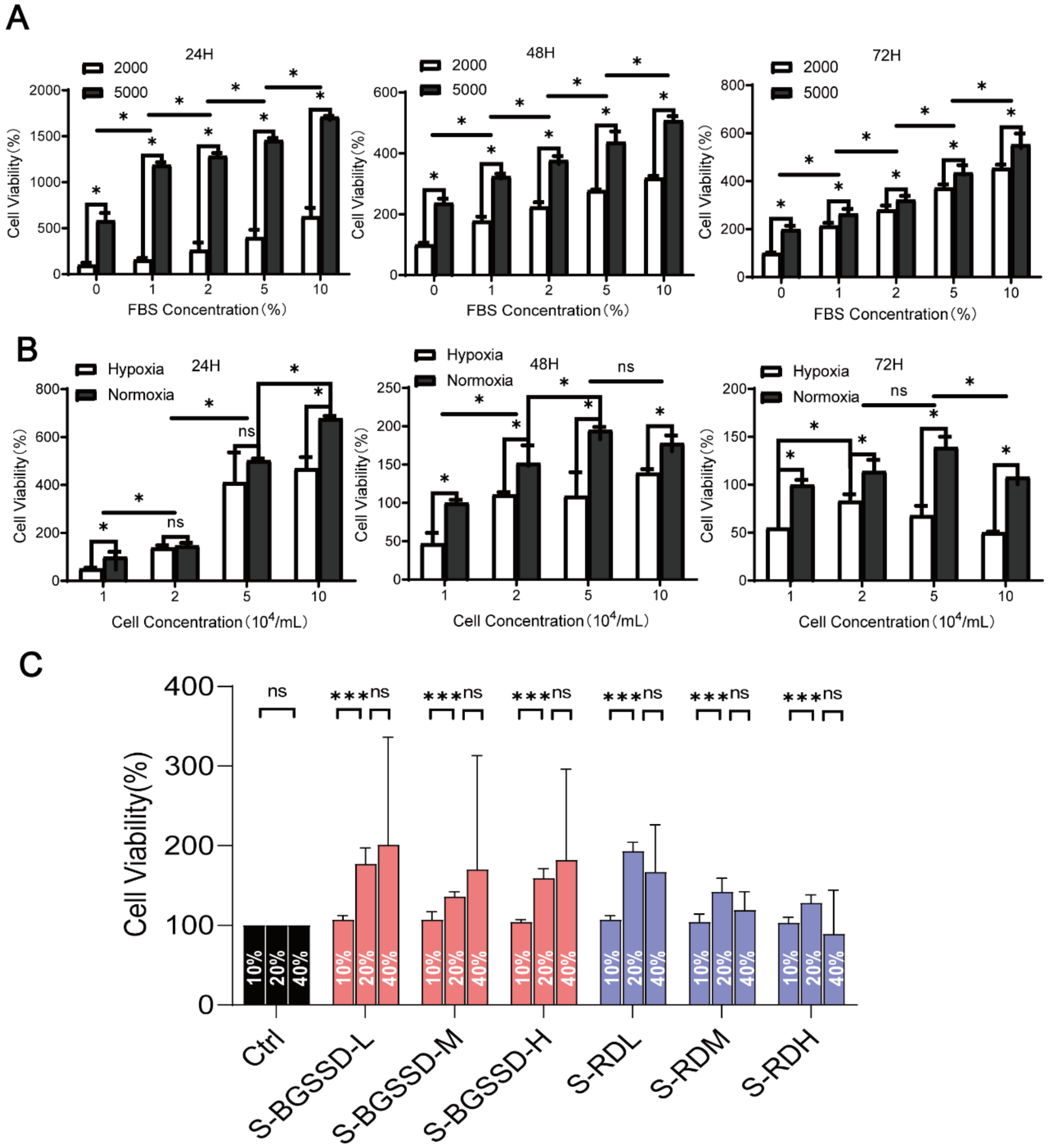

Figure S3. Results of MC3T3-E1 cells modeling and different concentrations of drug-containing serum intervening serum-starved MC3T3-E1 cells. **(A)** Cell viabilities of MC3T3-E1 cells under different concentrations of fetal bovine serum at different

intervened time. **(B)** Cell viabilities of MC3T3-E1 cells under different contents of oxygen at different intervened time. **(C)** The viability results of MC3T3-E1 cells intervened by different concentrations of drug-containing serum of BGSSD and Rhizoma Drynariae. The results are presented as the mean ±s.e.m., $^{*}P<0.05$, $^{**}P<0.01$, $^{***}P<0.001$. NS, no significant.

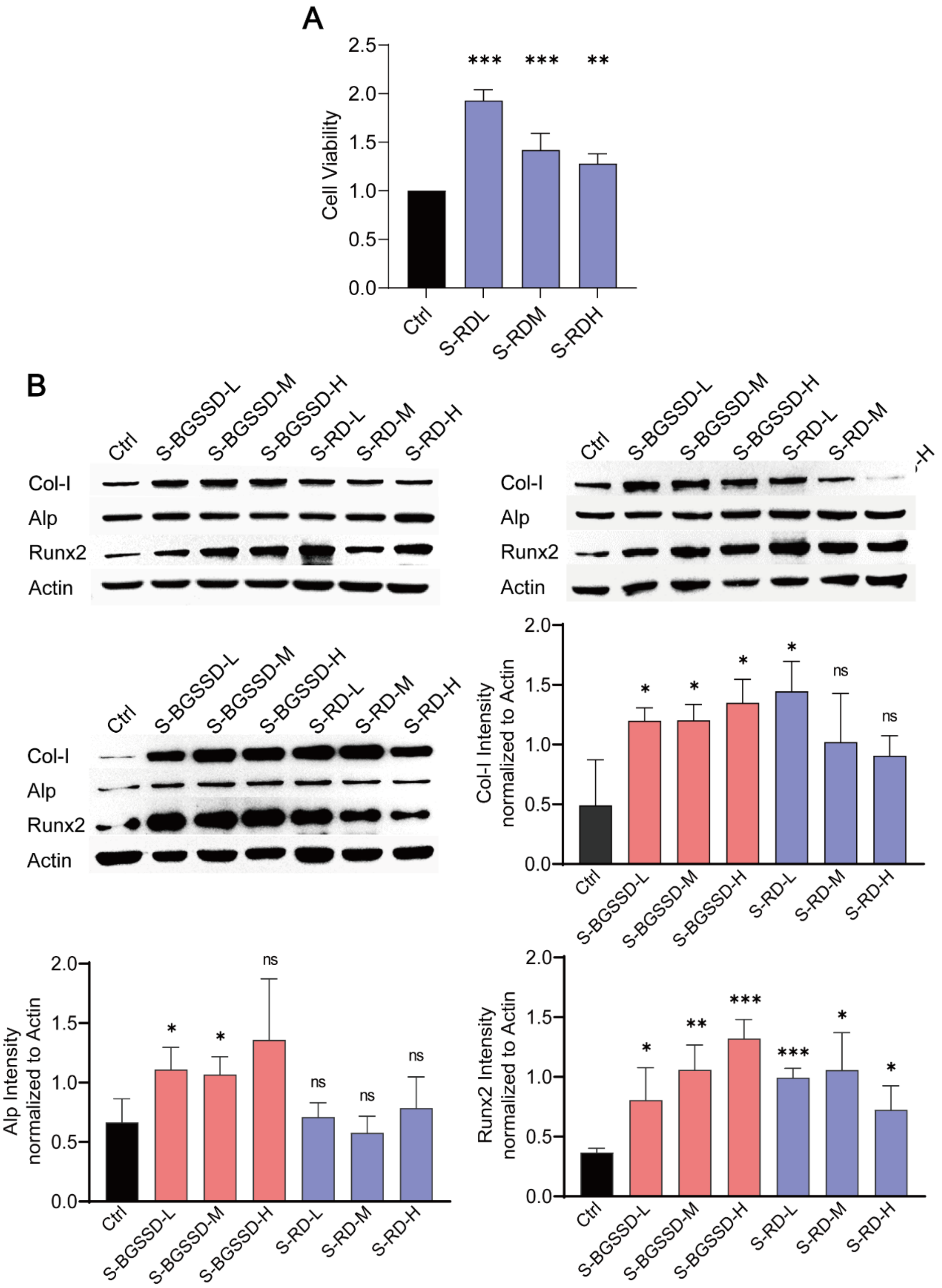

Figure S4. The results of drug-containing serum of Rhizoma Drynariae intervening in

the proliferation and differentiation of serum deprivation-induced MC3T3-E1 cells. **(A)** MTS assay analysis of the viability of MC3T3-E1 cells dealt with drug-containing serum of low, medium, and high-dose of Rhizoma Drynariae (in order to eliminate the influence of rat serum on cells, the Ctrl was treated with drug-free serum of rats). **(B)** Western blot and quantificational analysis of Col-I, ALP and Runx2 in MC3T3-E1 cells. Asterisks denote the comparison to serum-starved cells treated with rats' blank serum (Ctrl). The intervening time of drug-containing serum of Rhizoma Drynariae was 48 hours, β-actin served as the internal control in all western blots. Data are presented as the mean ±s.e.m., $^{*}P<0.05$, $^{**}P<0.01$, $^{***}P<0.001$, n=3. NS, no significant.

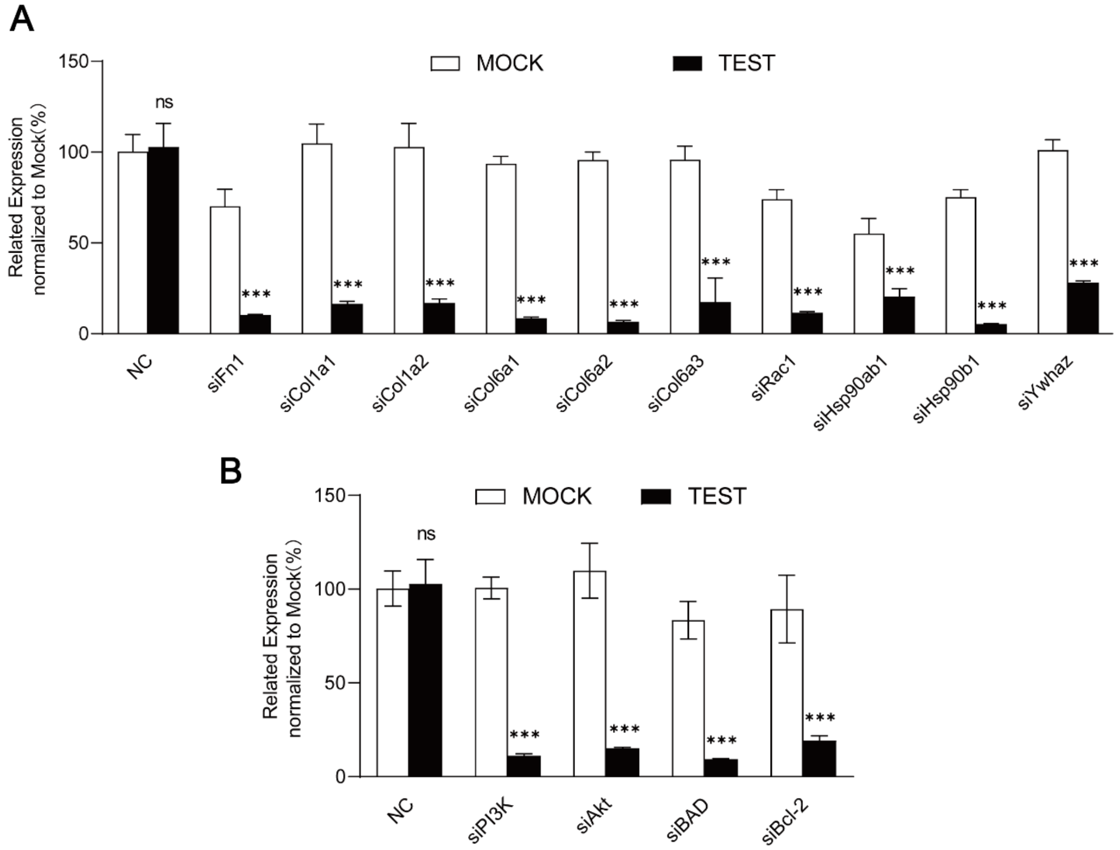

Figure S5. qRT-PCR assay to test the knock-down efficiency of targeting genes of PI3K-Akt pathway. **(A)** Each of the ten targeted genes (Fn1, Col1a1, Col1a2, Col6a1, Col6a2, Col6a3, Rac1, Hsp90ab1, Hsp90b1, and Ywhaz, except the Col6a3 and Ywhaz) was effectively knocked down by siRNA. **(B)** Each of the genes of PI3K-AKT and its downstream pathway, named PI3K, AKT, BAD, and Bcl-2, was effectively knocked down by siRNA. Data are presented as the mean ±s.e.m., $^{*}P<0.05$, $^{**}P<0.01$, $^{***}P<0.001$, n=4. NS, no significant.

Data availability statement: The datasets presented in this study can be found in online repositories, and the internal ID in PRIDE is "PXD030275".
