# Supplementary material for: Quantitative Proteomics Revealed the Pharmacodynamic Network of Bugu Shengsui Decoction Promoting Osteoblast Proliferation
Source: Front Endocrinol (Lausanne). 2022 Jan 25;12:833474. doi: 10.3389/fendo.2021.833474 (PMC8822948; doi:10.3389/fendo.2021.833474)

Dataset 2.

The raw data of ALP, Runx2, and Col-I expression results of MC3T3-E1 cells intervened by Bugu Shengsui Decoction and Rhizoma Drynariae. The results of 3 repeated biological experiments are displayed. The name of each sample is marked above the image, and the protein names are marked on the left of the image, with the molecular weight on the right.

Statement: In the raw data of "Dataset 2", we provide all the original results the full scans of the entire original gels.

The first template of new repetition-Alp

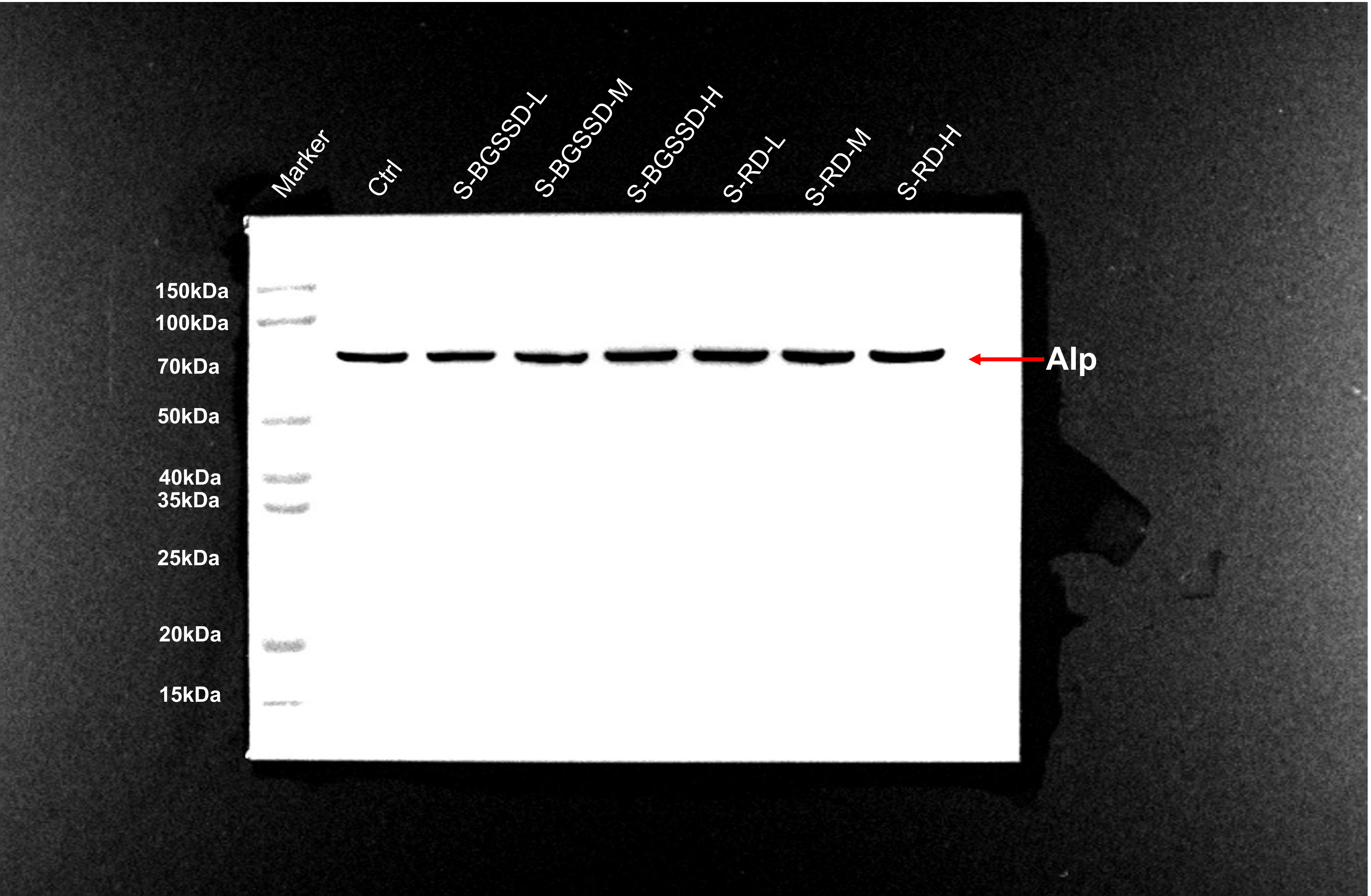

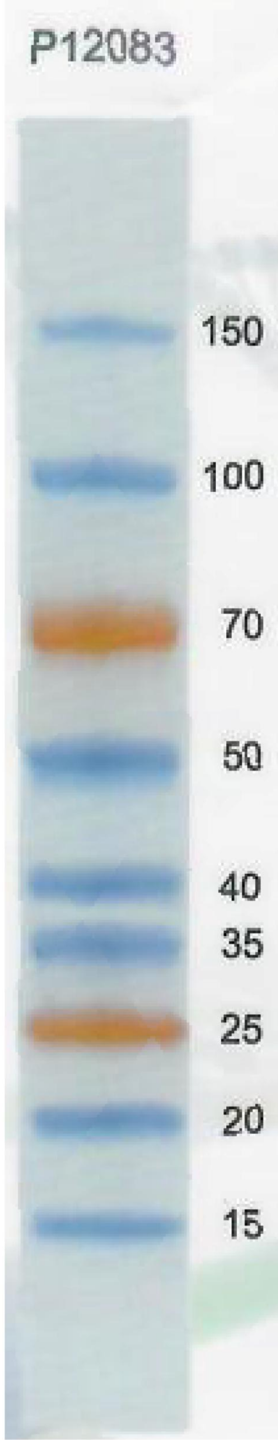

The second template of new repetition-Alp

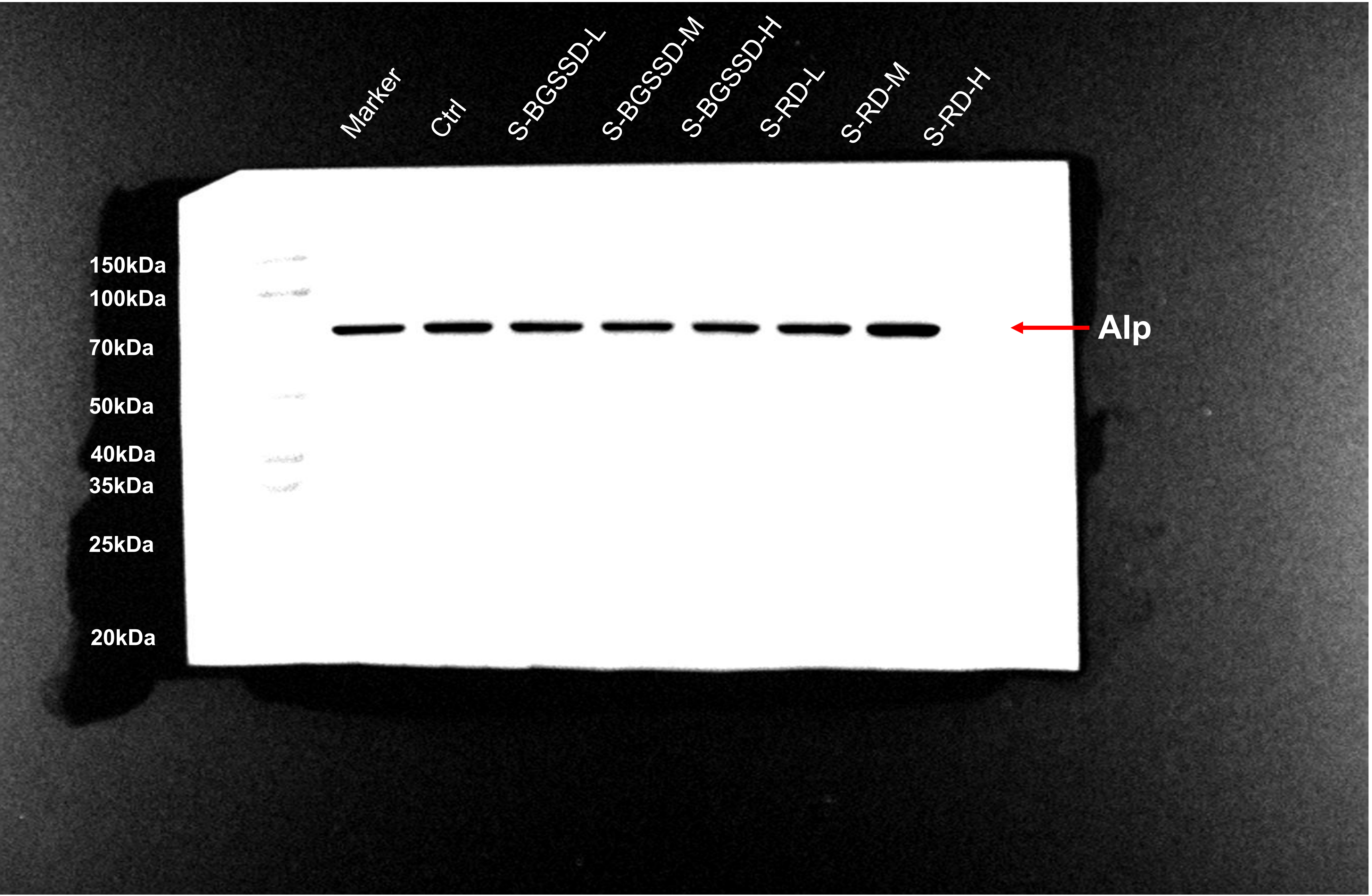

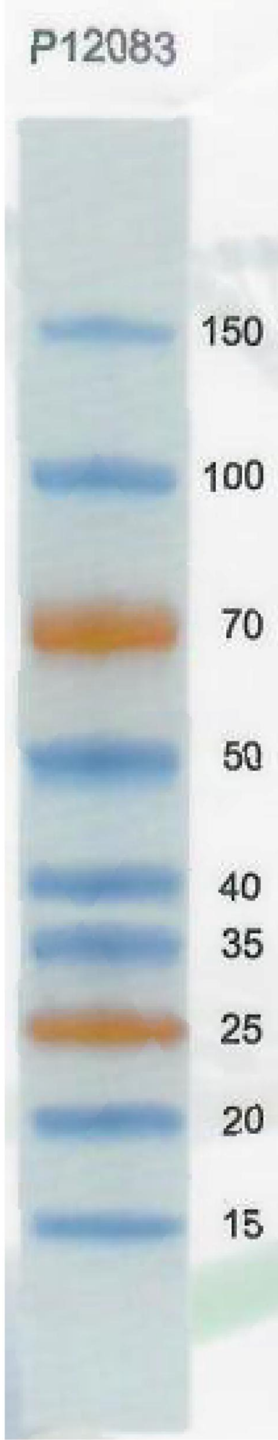

The third template of new repetition-Alp

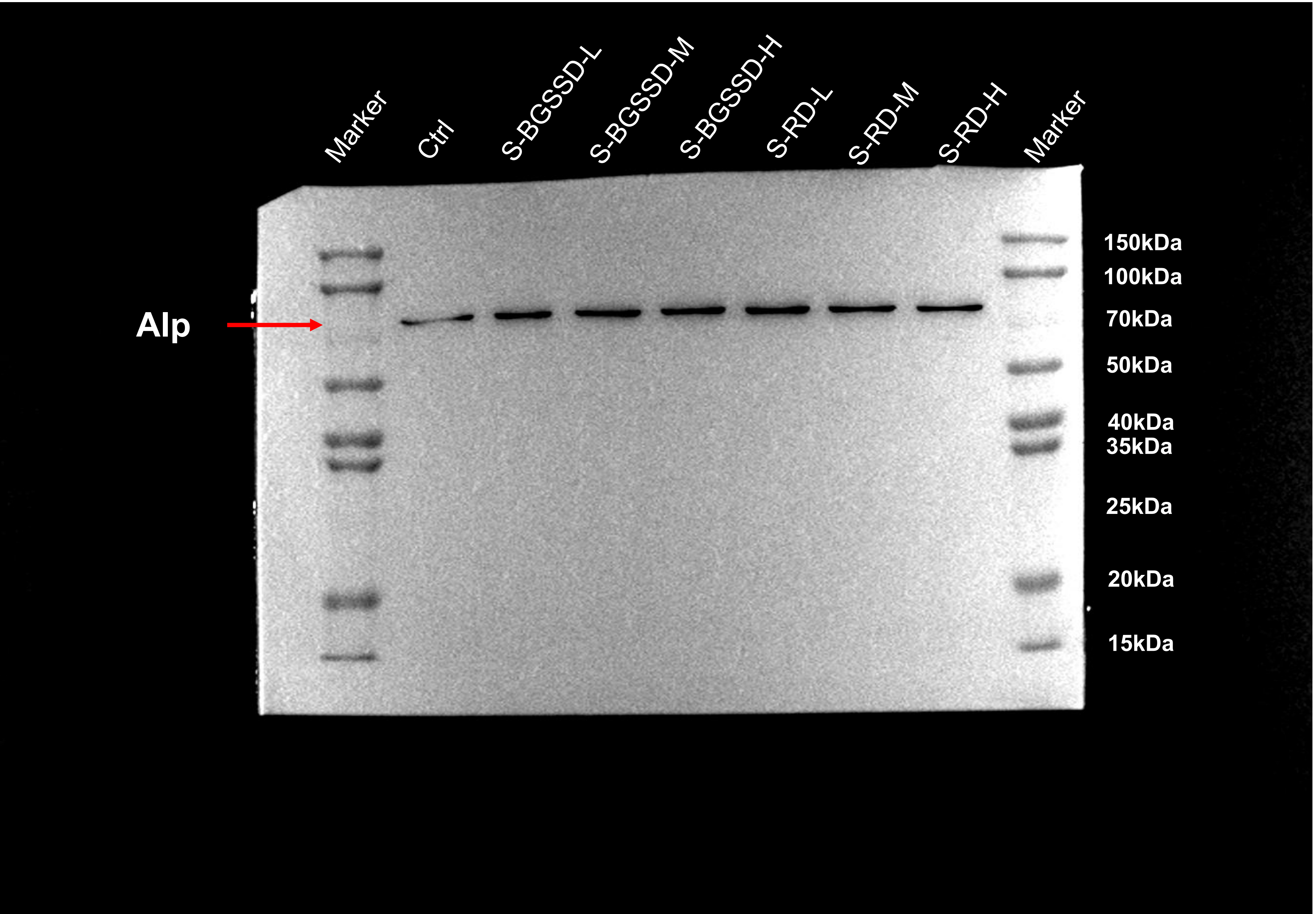

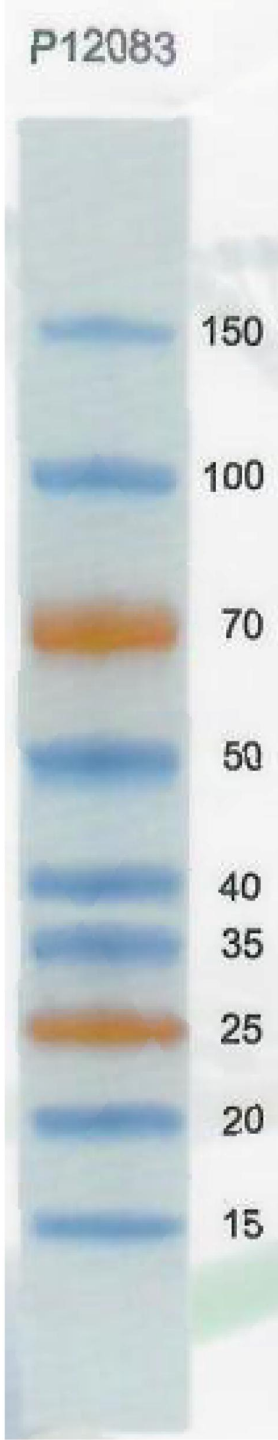

The first template of new repetition-Col-I

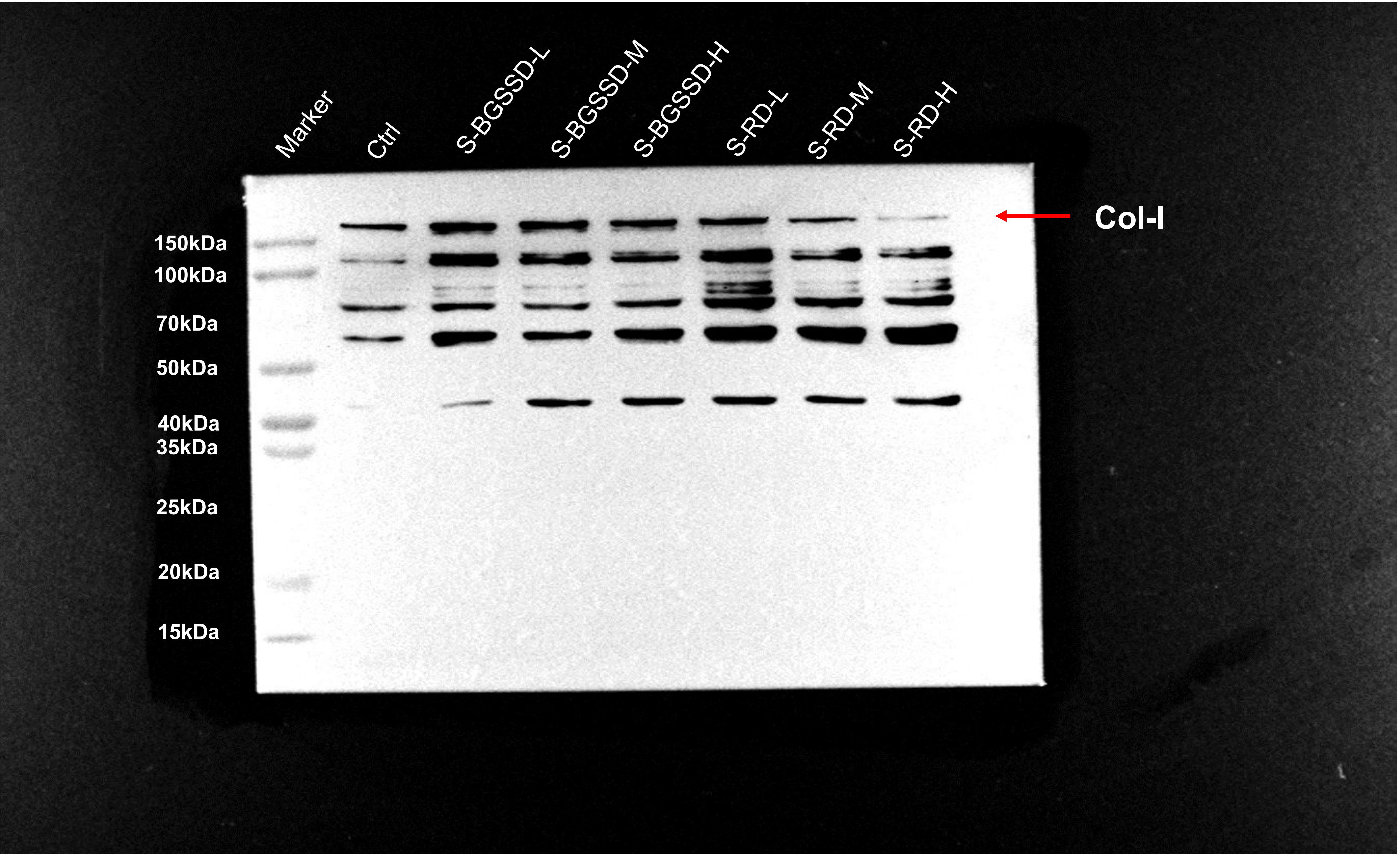

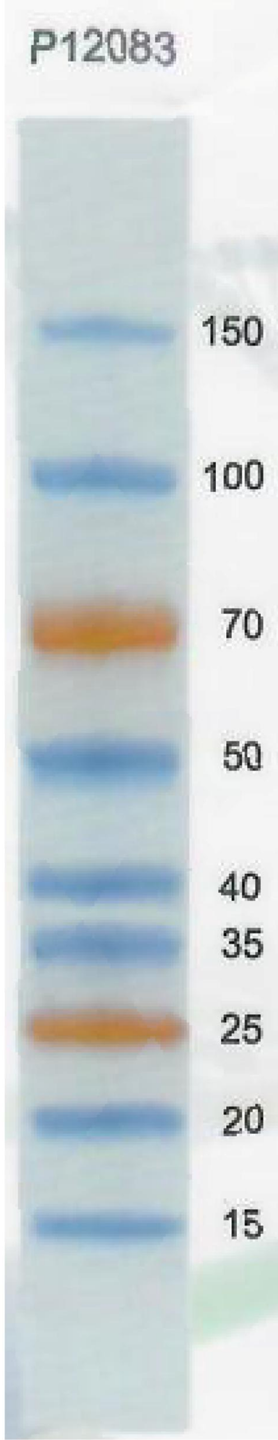

The second template of new repetition-Col-I

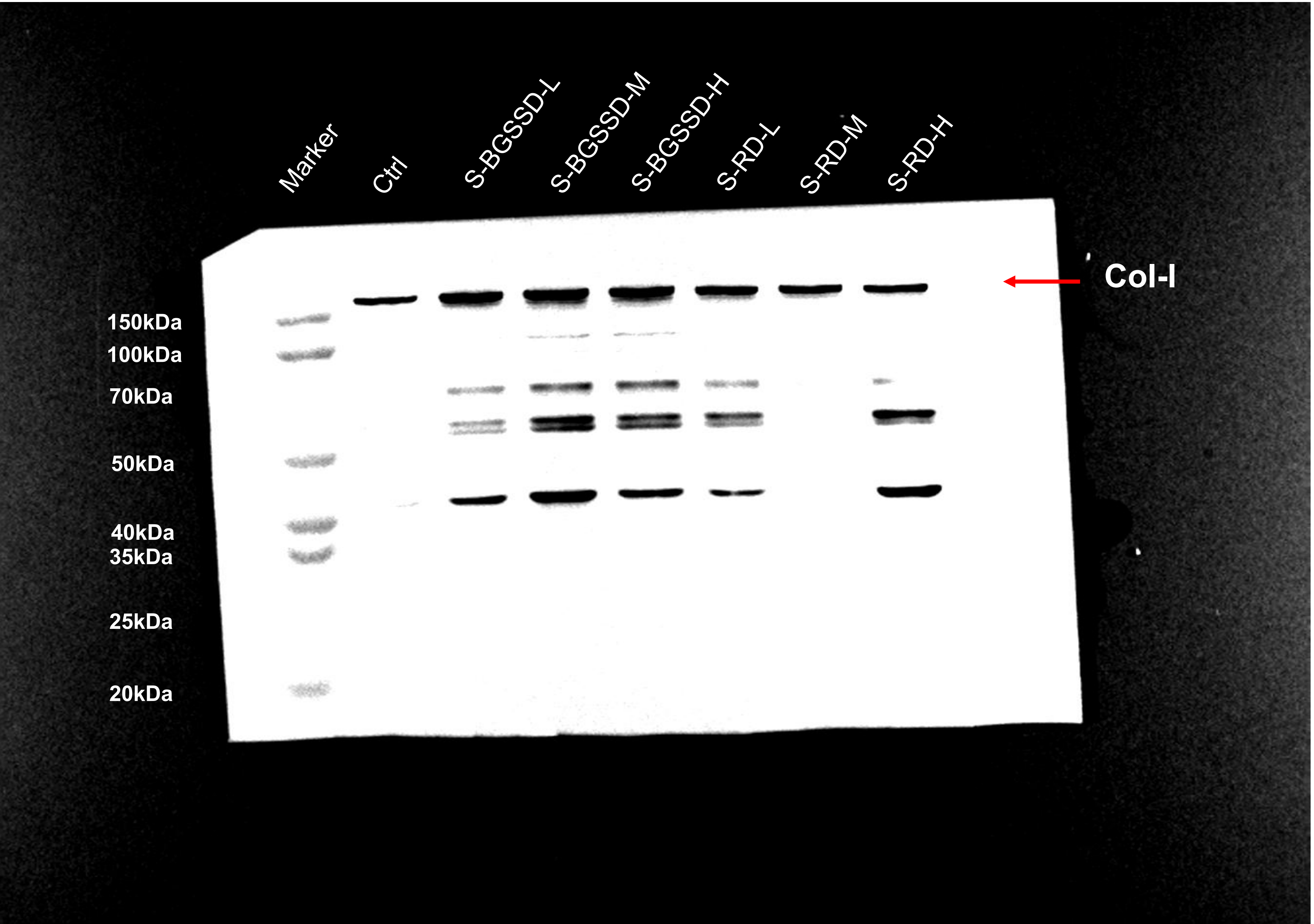

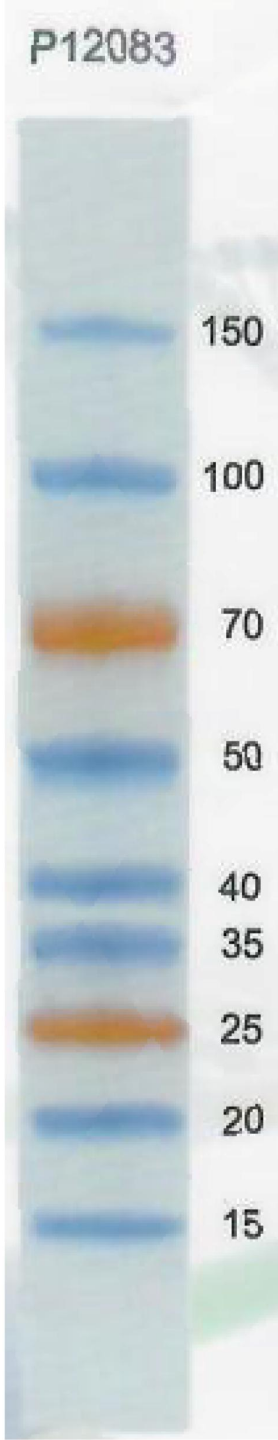

The third template of new repetition-Col-I

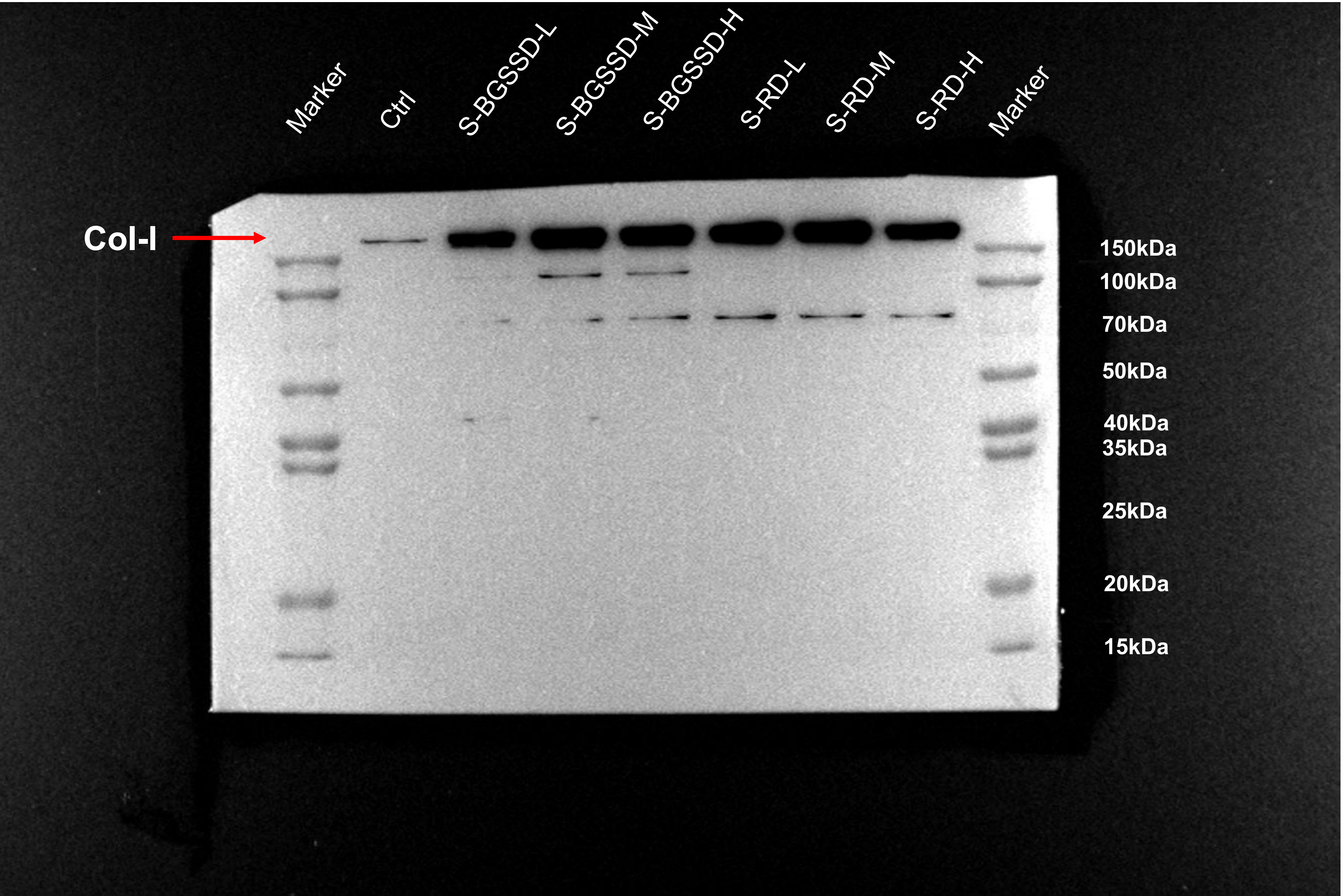

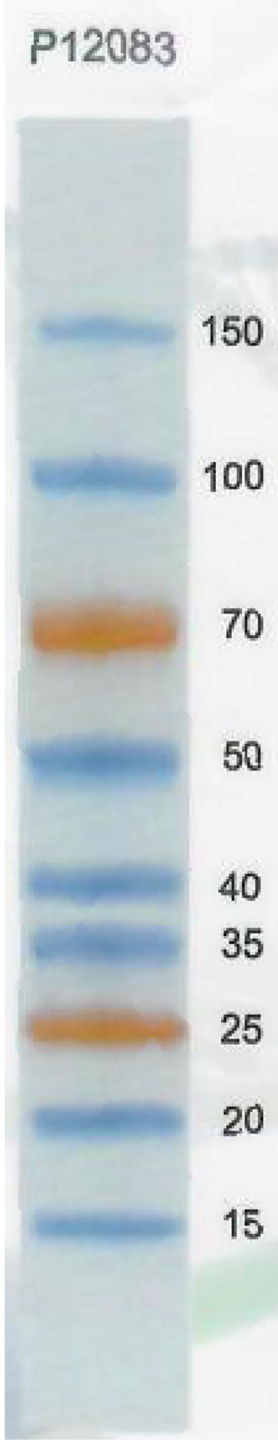

The first template of new repetition-Runx2

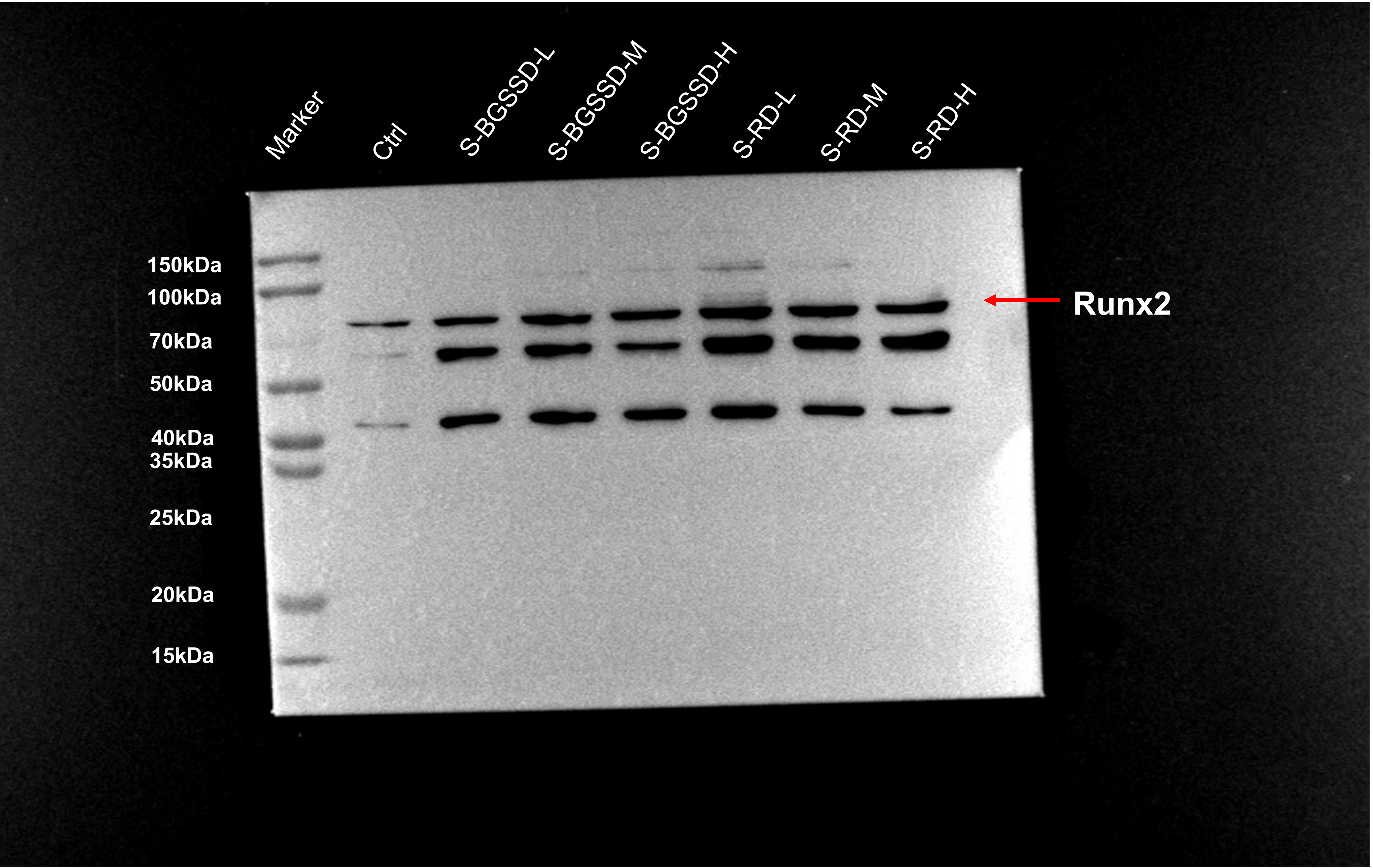

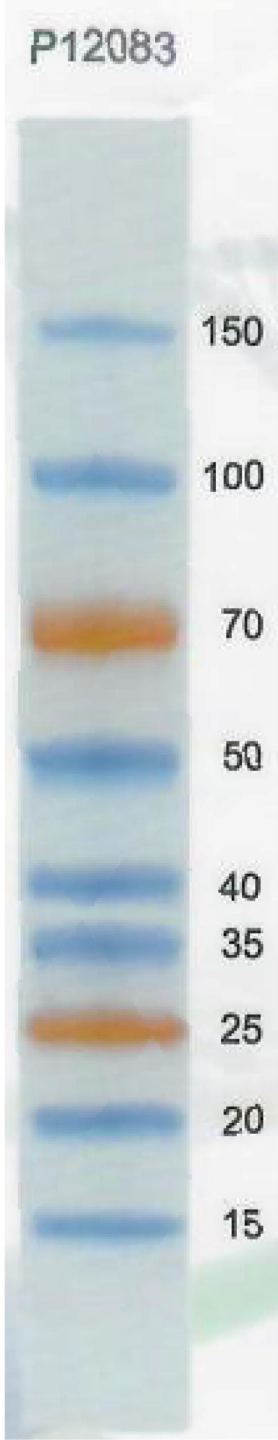

The second template of new repetition-Runx2

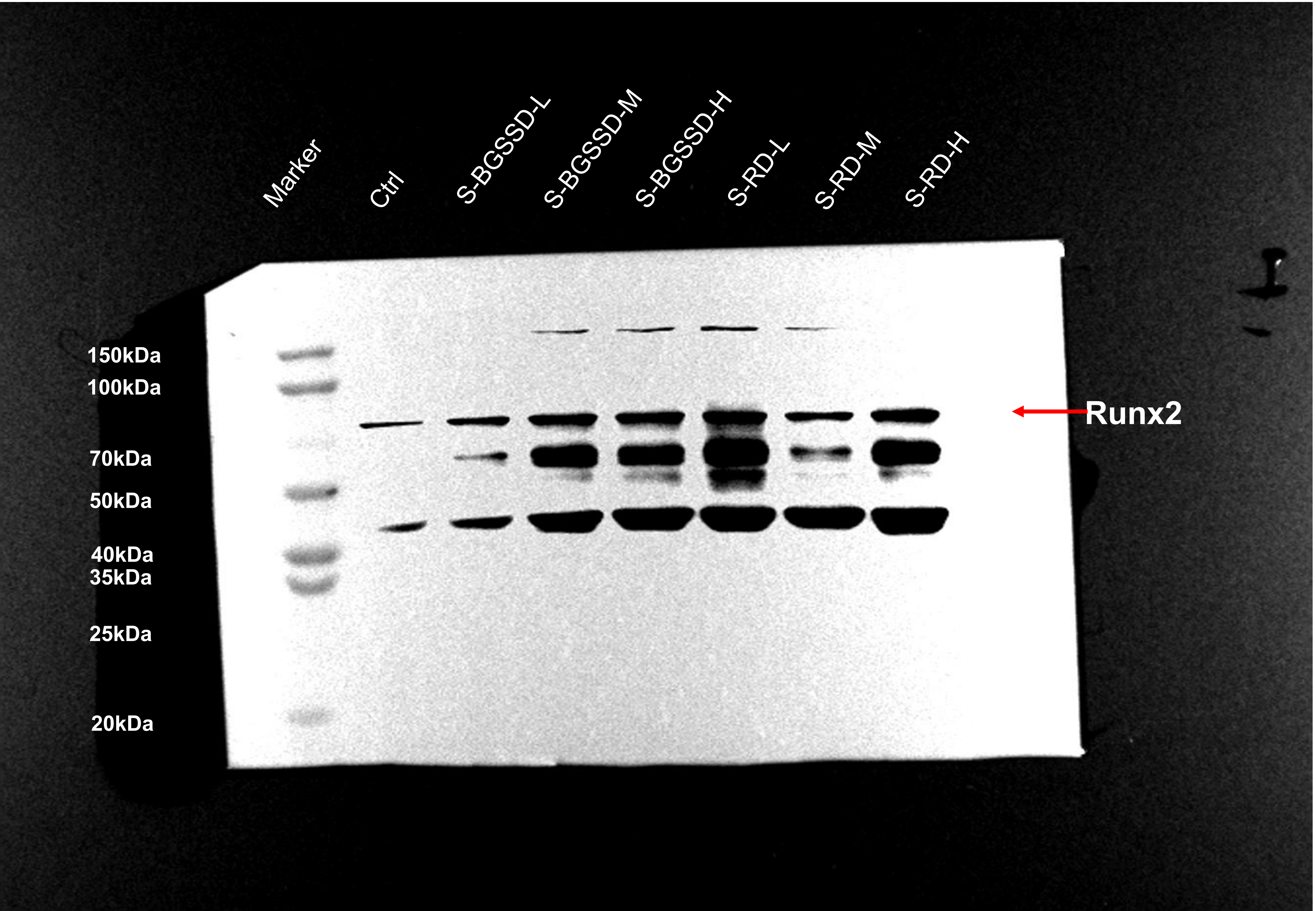

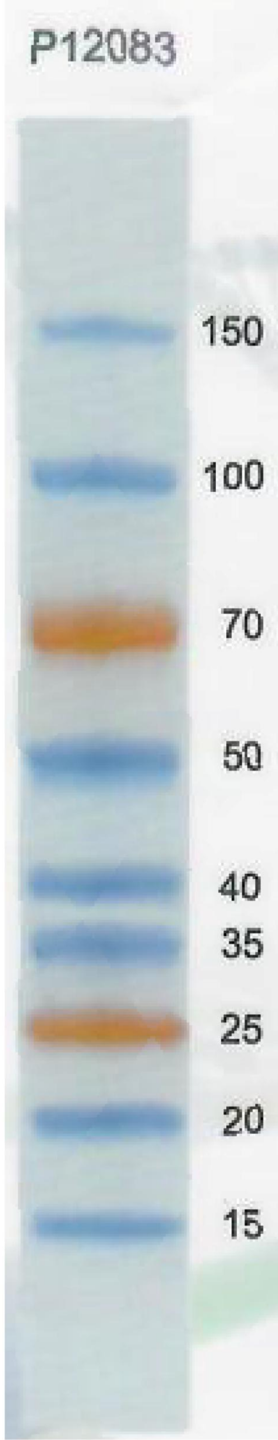

The third template of new repetition-Runx2

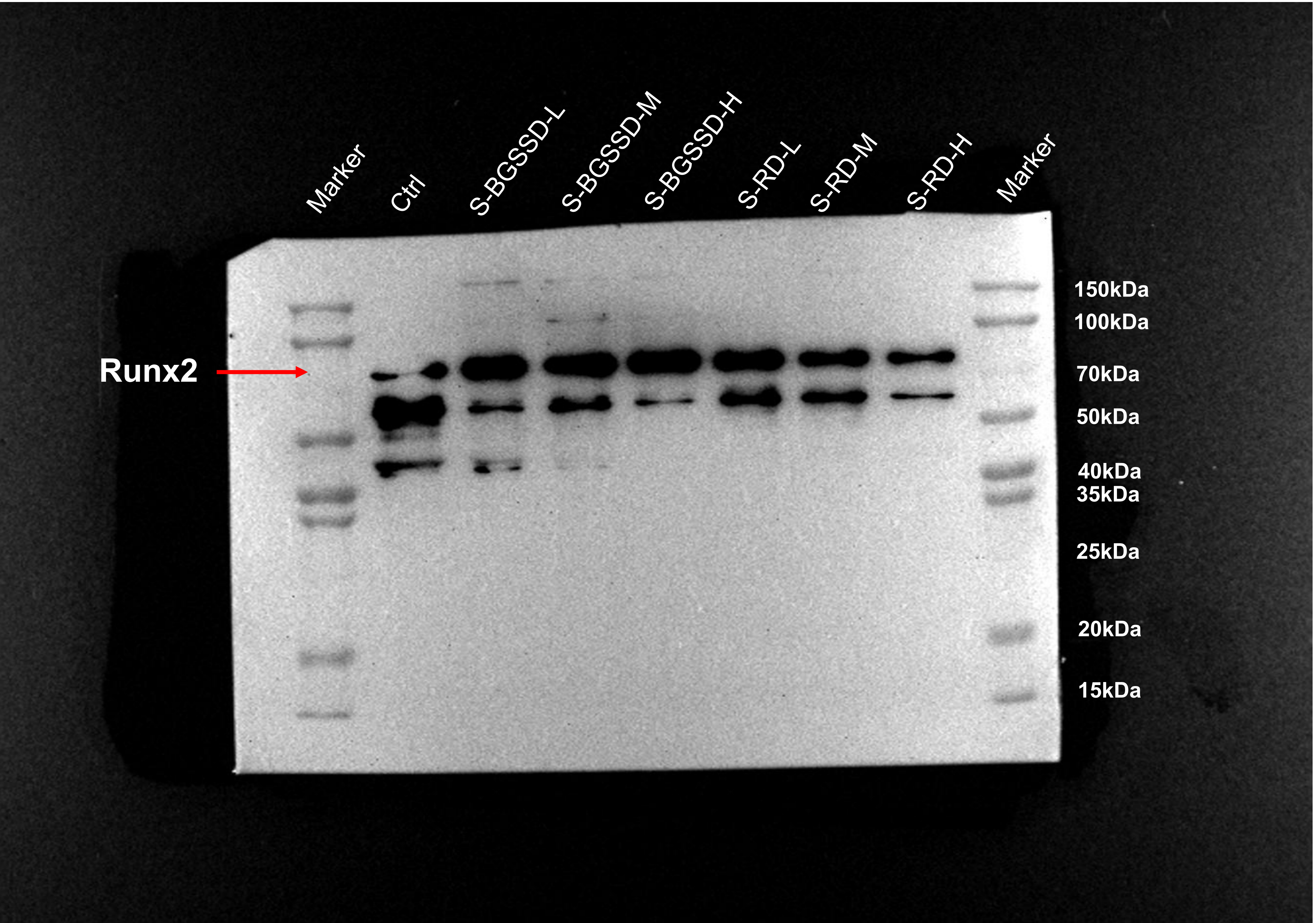

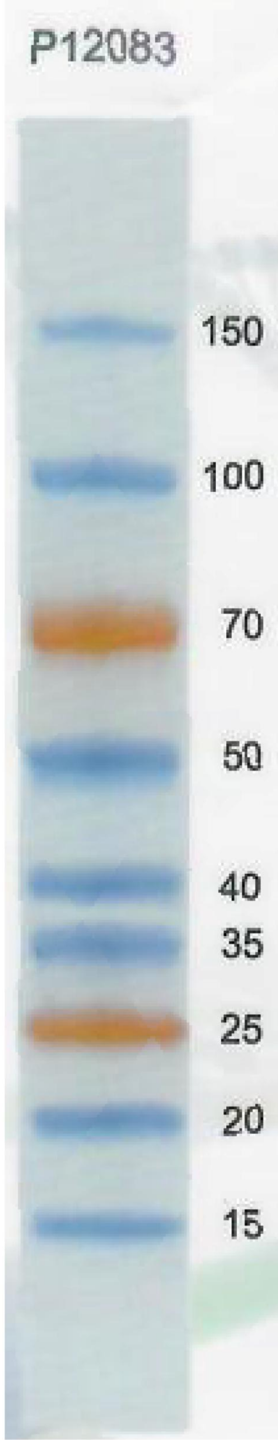

The first template of new repetition-β-Actin

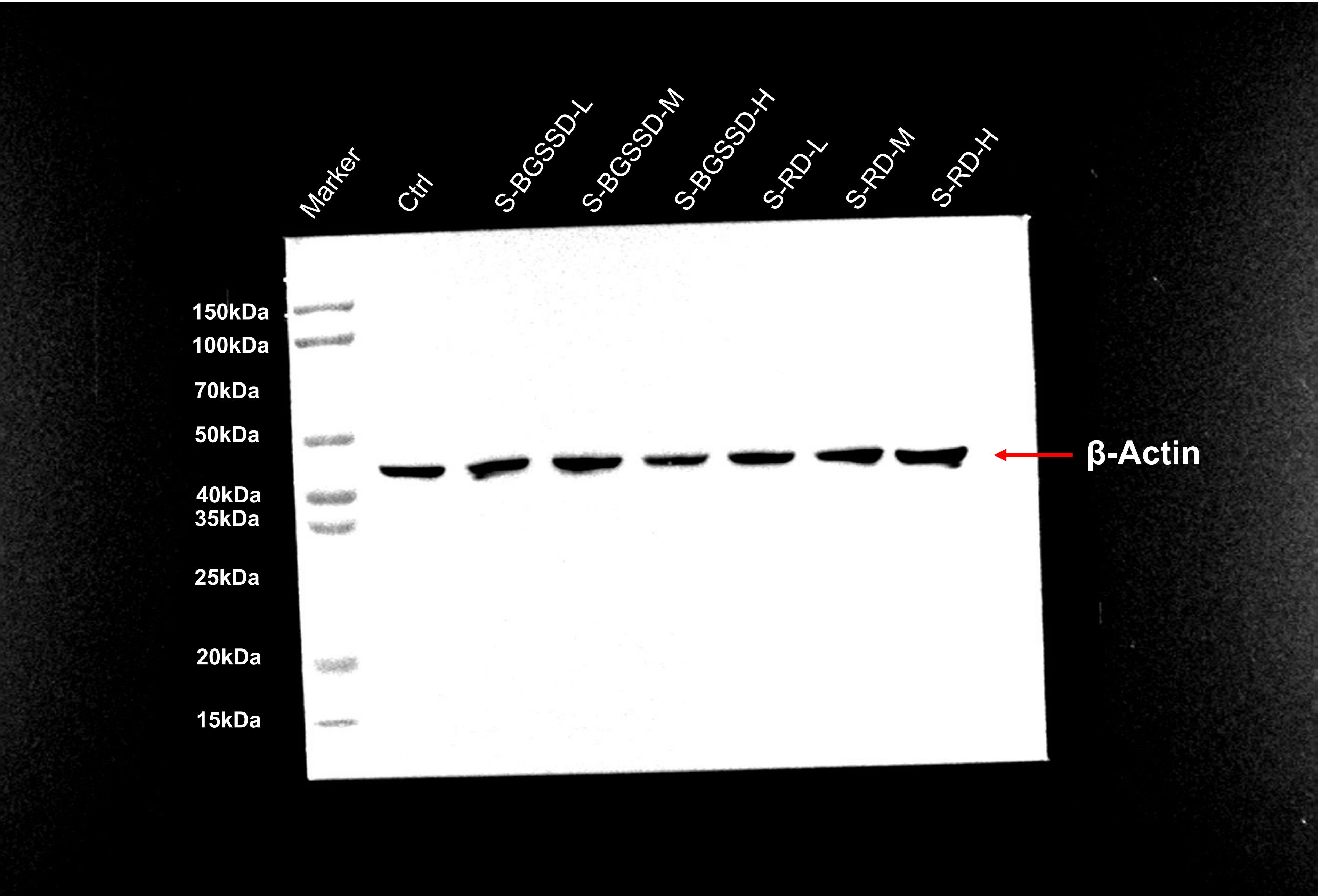

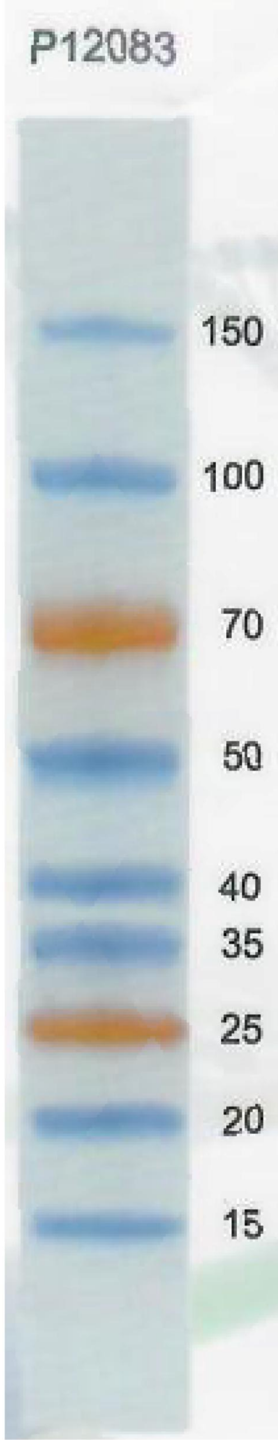

The first template of new repetition-β-Actin

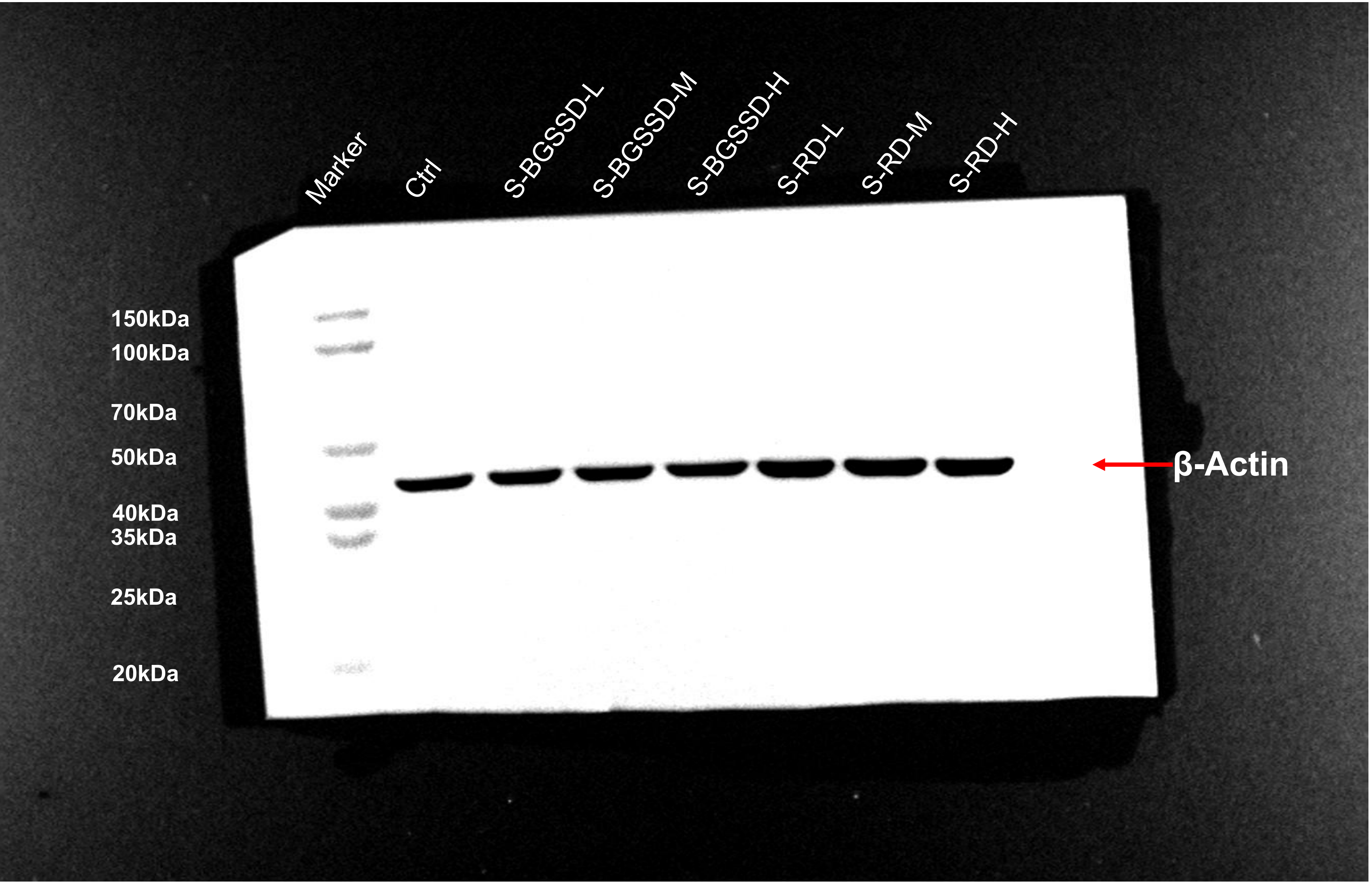

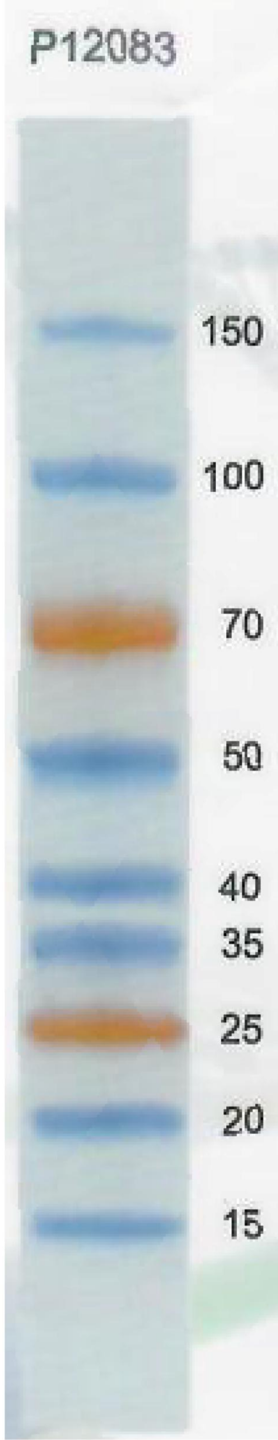

The third template of new repetition-β-Actin

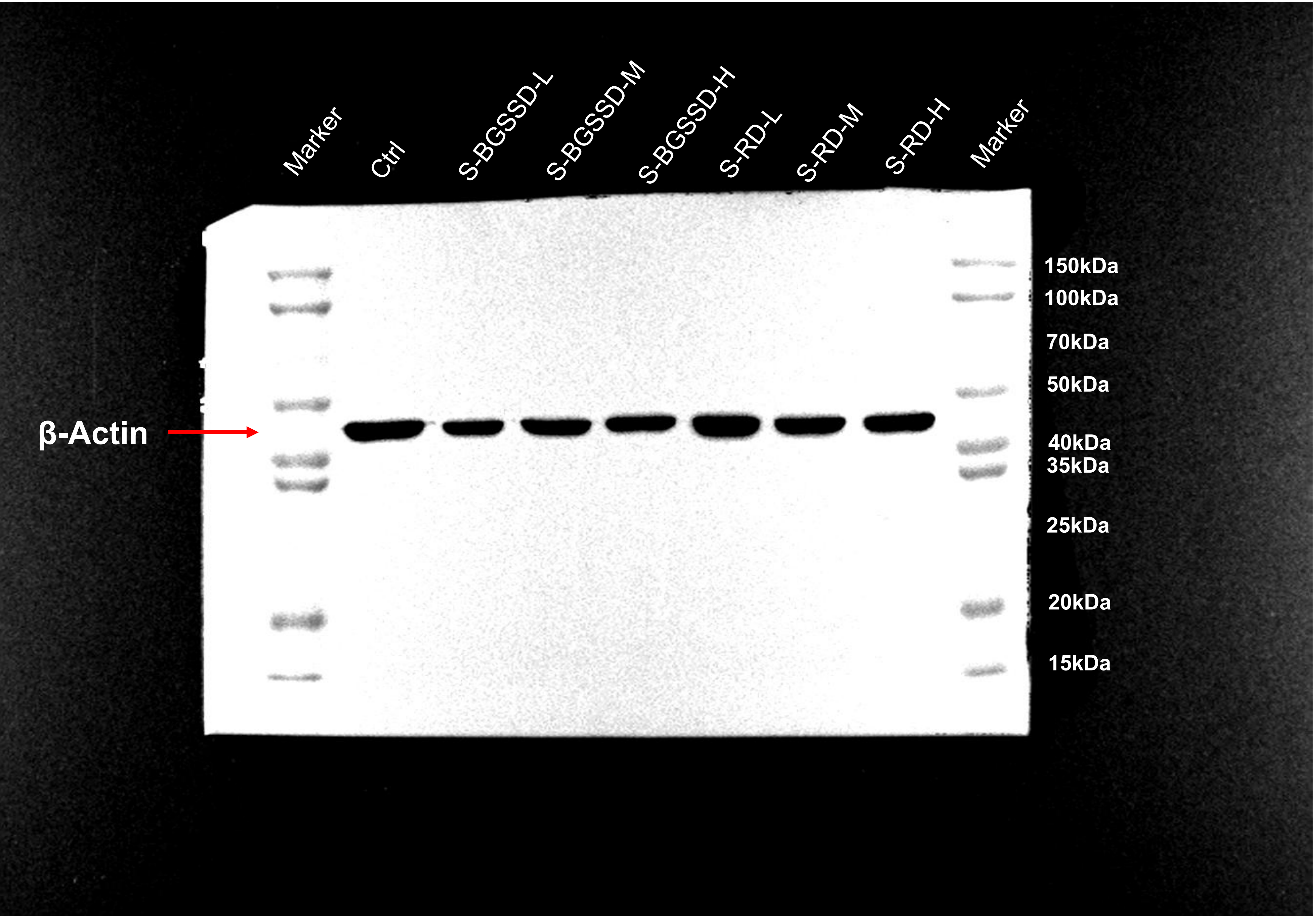

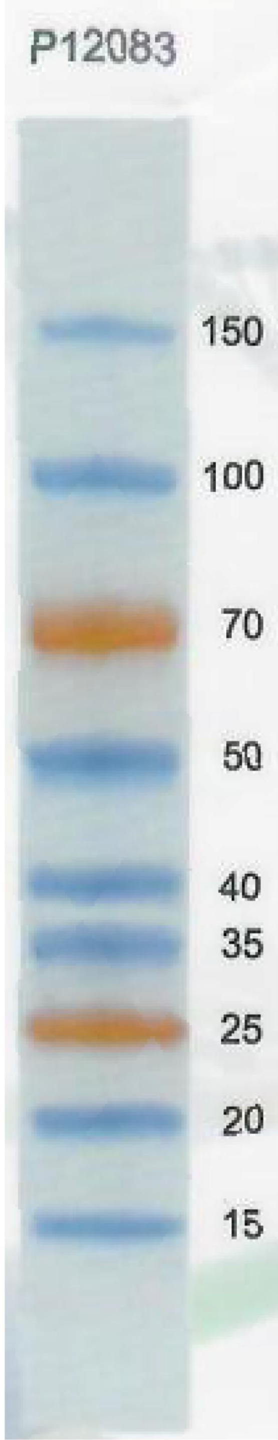

The first template of new repetition

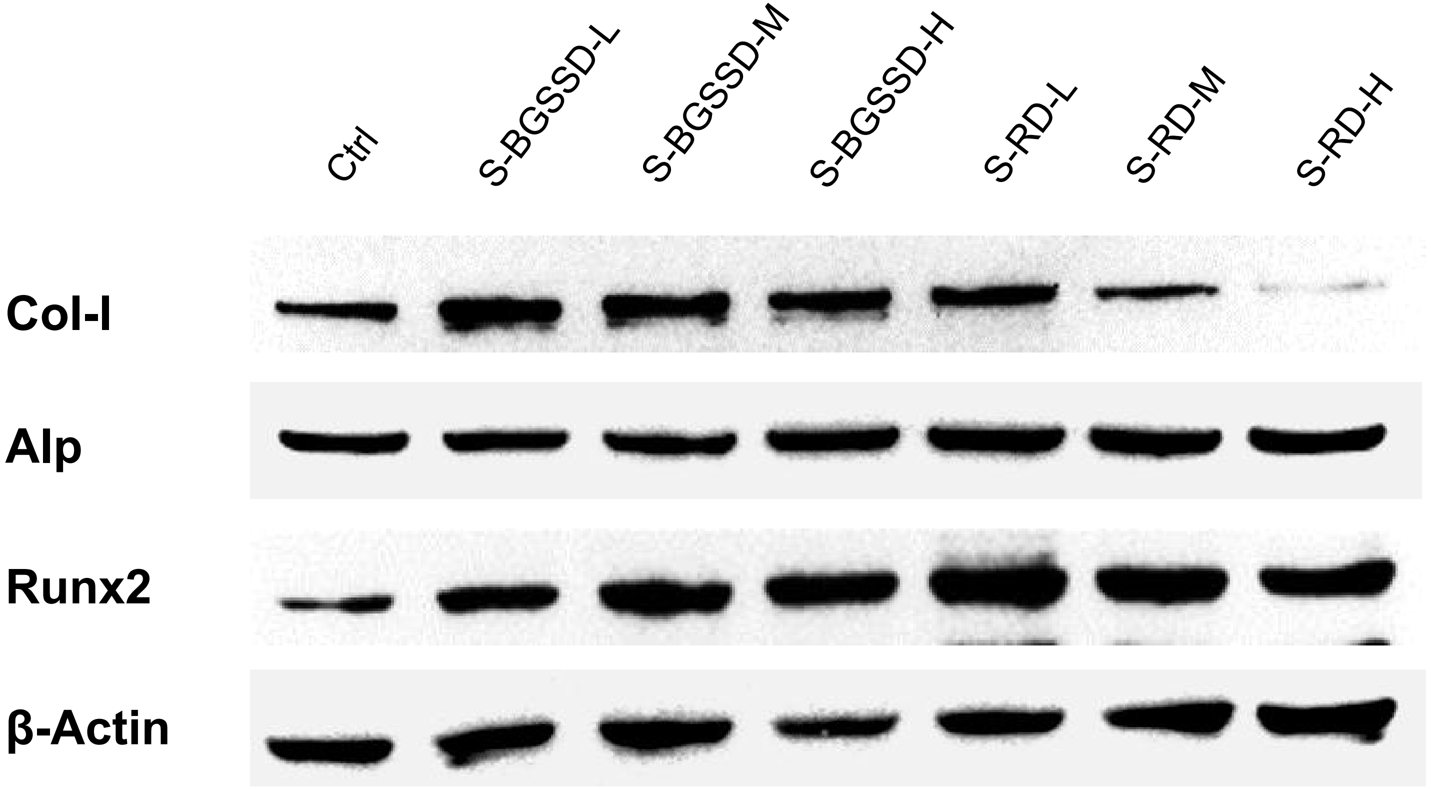

The second template of new repetition

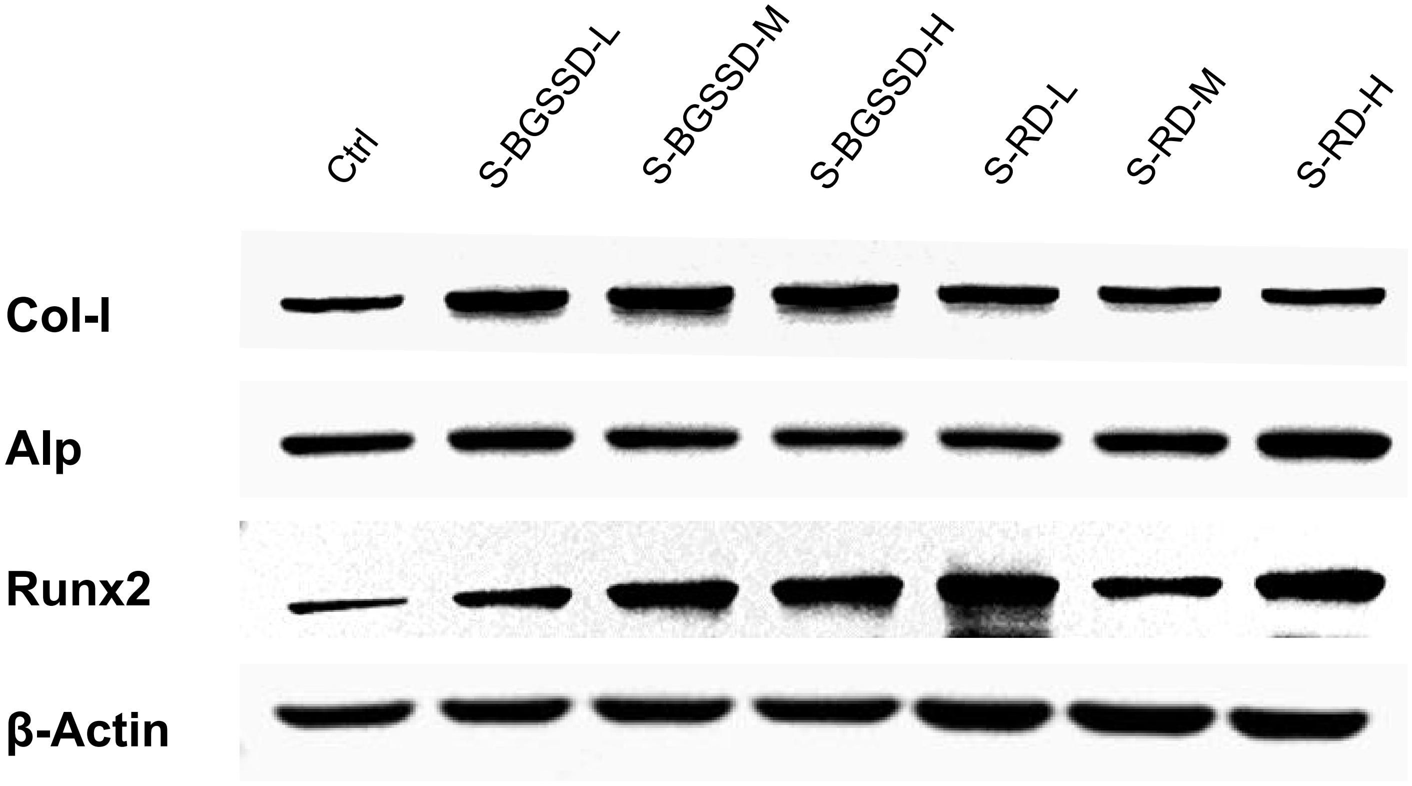

The third template of new repetition

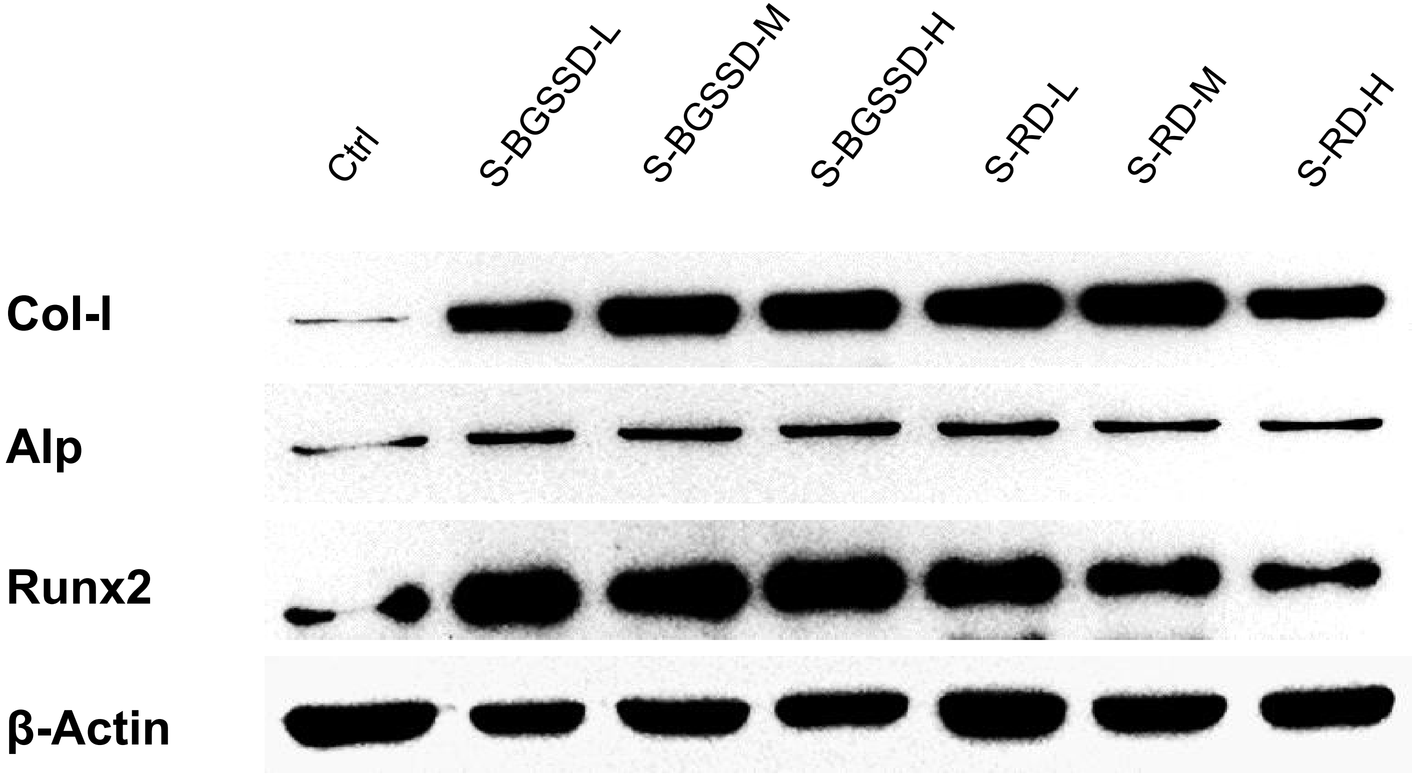

Supplement: Supplementary file 2 [file DataSheet_1.zip › Dataset 2.PDF]
