# Supplementary material for: Quantitative Proteomics Revealed the Pharmacodynamic Network of Bugu Shengsui Decoction Promoting Osteoblast Proliferation
Source: Front Endocrinol (Lausanne). 2022 Jan 25;12:833474. doi: 10.3389/fendo.2021.833474 (PMC8822948; doi:10.3389/fendo.2021.833474)
Supplement: Supplementary file 2 [file DataSheet_1.zip › Dataset summary.DOCX]

**Dataset 1.**

**The original results of all parameters in osteoporotic rats treated with different doses of Bugu Shengsui Decoction and Rhizoma Drynariae.** The bone metabolism indicators’ results such as serum ALP, calcium, and phosphate are listed in “Raw Data 1”. The results of bone mineral density are presented in “Raw Data 2”. Bone tissue morphology’s results including the ratio of bone volume to tissue volume (BV/TV), trabecular thickness (Tb.Th), trabecular number (Tb.N), trabecular separation (Tb.Sp) and structural model index (SMI), are showed in “Raw Data 3”. Bone tissue biomechanical outcomes such as maximum load, flexural strength, and elastic modulus are displayed in “Raw Data 4”. All parameters contain 6 outcomes in each group.

**Dataset 2.**

**The raw data of ALP, Runx2, and Col-I expression results of MC3T3-E1 cells intervened by Bugu Shengsui Decoction and Rhizoma Drynariae.** The results of 3 repeated biological experiments are displayed. The name of each sample is marked above the image, and the protein names are marked on the left of the image, with the molecular weights on the right.

**Statement:** In the raw data of “Dataset 2”, we provide all the original results of full scans of the entire original gels.

**Dataset 3.**

**The list of differential proteins of specific pharmacodynamic network affected by BGSSD identified through quantitative proteomics experiments.** The list contains protein original results of three replicates in “Raw Data1”, “Raw Data2”, and “Raw Data3”. The ratios (Light vs Heavy) of proteins from all the three templates are listed in “Selected Data”, and the average quantitative ratios of Bugu Shengsui Decoction (Heavy vs Light) are shown in the last column. The data of Bugu Shengsui Decoction are filtered with a ratio of 1.5, and 190 proteins meet the criteria, that are used for GO analysis. The ten proteins showed in “Targets” are from the functional cluster “PI3K-Akt signaling pathway” as shown in Fig. 4E.
